# Heritability Analyses Uncover Shared Genetic Effects of Lung Function and Change over Time

**DOI:** 10.3390/genes13071261

**Published:** 2022-07-15

**Authors:** Donghe Li, Woojin Kim, Jahoon An, Soriul Kim, Seungku Lee, Ahra Do, Wonji Kim, Sanghun Lee, Dankyu Yoon, Kwangbae Lee, Seounguk Ha, Edwin K. Silverman, Michael Cho, Chol Shin, Sungho Won

**Affiliations:** 1 Interdisciplinary Program in Bioinformatics, Seoul National University, Seoul 08826, Korea; lidonghe111@gmail.com (D.L.); holy30000@gmail.com (A.D.); 2 Department of Medicine (Biomedical Genetics), Boston University School of Medicine, Boston, MA 02118, USA; 3 Department of Internal Medicine and Environmental Health Center, School of Medicine, Kangwon National University, Chuncheon 24341, Korea; wjkim47@gmail.com; 4 Department of Public Health Science, Graduate School of Public Health, Seoul National University, Seoul 08826, Korea; mudblood.ev@gmail.com; 5 Institute for Human Genomic Study, College of Medicine, Korea University, Seoul 136701, Korea; soriul82@gmail.com (S.K.); leeseungku@gmail.com (S.L.); 6 Channing Division of Network Medicine and Division of Pulmonary and Critical Care Medicine, Brigham and Women’s Hospital, Boston, MA 02115, USA; rewki@channing.harvard.edu (W.K.); ed.silverman@channing.harvard.edu (E.K.S.); remhc@channing.harvard.edu (M.C.); 7 Department of Medical Consilience, Graduate School, Dankook University, Yongin 16890, Korea; sanghunlee73@gmail.com; 8 Division of Allergy and Respiratory Disease Research, Department of Chronic Disease Convergence Research, National Institute of Health, Korea Disease Control and Prevention Agency, Cheongju 28159, Korea; dyoon@korea.kr; 9 Korea Medical Institute, Seoul 03173, Korea; zenithkb@kmi.or.kr (K.L.); suha@kmi.or.kr (S.H.); 10 Division of Pulmonary Sleep and Critical Care Medicine, Department of Internal Medicine, Korea University Ansan Hospital, Ansan 15355, Korea; 11 Institute of Health and Environment, Seoul National University, Seoul 08826, Korea; 12 RexSoft Inc., Seoul 08826, Korea

**Keywords:** single nucleotide polymorphism, pulmonary function tests, likelihood functions, longitudinal study, heritability

## Abstract

Genetic influence on lung functions has been identified in previous studies; however, the relative longitudinal effects of genetic factors and their interactions with smoking on lung function remain unclear. Here, we identified the longitudinal effects of genetic variants on lung function by determining single nucleotide polymorphism (SNP) heritability and genetic correlations, and by analyzing interactions with smoking. Subject-specific means and annual change rates were calculated for eight spirometric measures obtained from 6622 Korean adults aged 40–69 years every two years for 14 years, and their heritabilities were estimated separately. Statistically significant (*p* < 0.05) heritability for the subject-specific means of all spirometric measures (8~32%) and change rates of forced expiratory volume in 1 s to forced vital capacity ratio (FEV_1_/FVC; 16%) and post-bronchodilator FEV_1_/FVC (17%) were detected. Significant genetic correlations of the change rate with the subject-specific mean were observed for FEV_1_/FVC ($\rho_{g}$ = 0.64) and post-bronchodilator FEV_1_/FVC ($\rho_{g}$ = 0.47). Furthermore, post-bronchodilator FEV_1_/FVC showed significant heritability of SNP-by-smoking interaction ($h_{GXS}^{2}$ = 0.4) for the annual change rate. The GWAS also detected genome-wide significant SNPs for FEV_1_ (rs4793538), FEV_1_/FVC (rs2704589, rs62201158, and rs9391733), and post-bronchodilator FEV_1_/FVC (rs2445936). We found statistically significant evidence of heritability role on the change in lung function, and this was shared with the effects on cross-sectional measurements. We also found some evidence of interaction with smoking for the change of lung function.

## 1. Introduction

Lung function is usually assessed using spirometry, which reflects the physiological state of the lungs and airways [1]. Chronic obstructive pulmonary disease (COPD), characterised by airflow limitation as assessed by spirometric measurement, imposes a large burden in terms of disability and is a leading cause of death worldwide [2,3,4]. Several studies have investigated the genetics of lung function by estimating heritability and performing a genome-wide association study (GWAS). Family-based studies have provided evidence of significant genetic effects on lung function, with estimates of heritability ($h^{2}$) ranging from 0.28 to 0.52 for forced expiratory volume in 1 s (FEV_1_), 0.4 to 0.54 for forced vital capacity (FVC), and 0.24 to 0.45 for FEV_1_/FVC [5,6,7,8]. In addition, Zhou et al. estimated the single nucleotide polymorphism (SNP) heritability of FEV_1_ and FEV_1_/FVC in a non-Hispanic white cohort of smokers with and without COPD and found that both were approximately 0.37 [9].

Genome-wide association studies have identified specific SNPs associated with FEV_1_, FVC, and FEV_1_/FVC [10]. Several studies have detected genetic signals associated with lung functions, including variants in *KANSL1*, *HLA-DQ*, *NPNT*, *TET2*, *TSEN54*, and *HHIP*, in non-smokers, and *RBM19-TBX5* in heavy smokers [11,12,13,14,15,16]. However, these studies focused on cross-sectional lung function, whereas just a few studies have focused on the genetic effects underlying the rate of decline in lung function [17].

Epidemiologic and genetic investigations of pulmonary function require longitudinal observation of lung function trajectories. Although a few studies followed subjects for more than 10 years [17,18], to the best of our knowledge, none of these studies assessed heritability in unrelated subjects, or subjects with non-European ancestry. The advantage of using unrelated subjects to estimate heritability is that they do not suffer from confounding problems caused by epistatic interactions or shared environment, which are present in family and twin studies [19,20,21,22].

In the present study, we utilised data from the Korean Genome and Epidemiology Study (KoGES) cohort [23]. All independent subjects in the KoGES cohort were enrolled when they were approximately 40 years of age and followed for 14 years. Eight biennially measured lung functions were analysed in this study. Using KoGES cohort data, we evaluated the importance of genetic components on the subject-specific means and annual change rates of lung function. Specifically, we defined two different heritability estimation approaches for the subject-specific mean, and annual change rates of longitudinally observed lung function changes were based on the method of Yang et al. [24]. We also performed GWAS on these lung functions.

## 2. Results

### 2.1. Characteristics of Study Subjects

**Figure 1:**
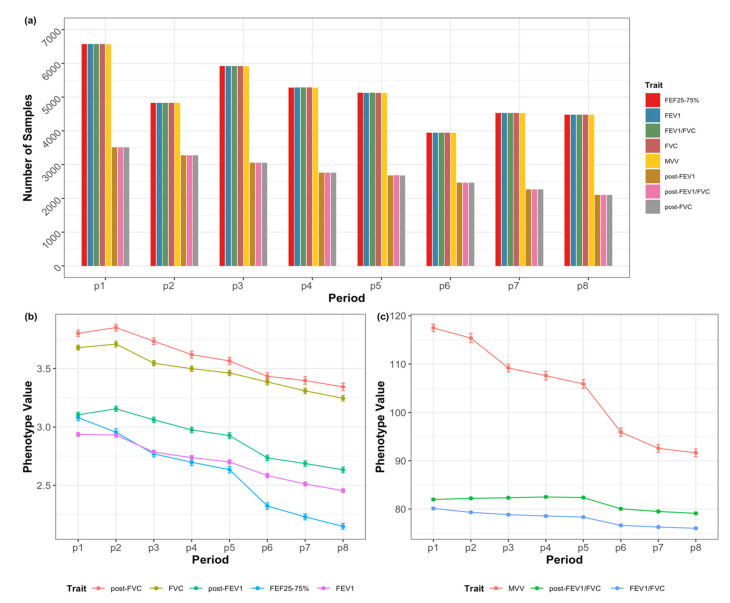
Number of samples used for analyzing the eight lung function traits and the mean observed values across eight periods. (**a**) Numbers of samples in each period (p1–p8). (**b**,**c**) Profile plot of the mean values of the eight traits in each period. Since the ranges of the traits differed, they are presented as two separate plots. FVC, forced vital capacity; FEV_1_, forced expiratory volume in 1 s; FEV_1_/FVC, forced expiratory volume in 1 s to forced vital capacity ratio; FEF 25–75%, the average forced expiratory flow during the mid (25–75%) portion of the FVC; MVV, maximal voluntary ventilation; post-FVC, post-bronchodilator test for FVC; post-FEV_1_, post-bronchodilator test for FEV_1_; post- FEV_1_/FVC, post-bronchodilator test for FEV_1_/ FVC ratio.

Study subjects were obtained from the Ansan (urban community) and Ansung (rural community) cohorts, which were followed up biennially. Each subject underwent a maximum of eight measurements. The numbers of observed measurements of the eight lung functions at each period are shown in Figure 1a and Supplementary Table S1. Notably, the sample size for the post-bronchodilator tests post-FVC, post-FEV_1_, and post-FEV_1_/FVC was small; these test results were available only from subjects in Ansung. The average profile plots of the eight lung functions at each period showed a decreasing trend for all lung functions (Figure 1b,c).

The statistical analyses required estimates of the mean and annual change rates for each subject, for which we included 6622 participants (3569 Ansan and 3053 Ansung) who were followed up ≥3 times (Table 1). Since some of the participants had a missing smoking status, 5104 participants were analysed in the smoking-related studies (3009 never-smokers and 2095 ever-smokers). In addition, 3,352,722 SNPs were considered in the analysis after quality control and imputation.

### 2.2. Heritability Estimates of Subject-Specific Means and Annual Change Rates

To estimate the importance of genetic determinants for the eight lung function traits, we calculated the subject-specific mean (${\hat{\beta }}_{0}$) and annual change rate (${\hat{\beta }}_{1}$) of each trait. Supplementary Table S2 shows the statistics of subject-specific mean and annual change rate. The estimated mean value of FVC across the eight periods was 3.47 and the mean value of FEV_1_, across the eight periods, was 2.7. Both of the estimated mean values of these two traits were in the range of average normal values, even though both were slightly lower. The mean of FEV_1_/FVC ratio was 77.97%, which is also within the normal range above the cutoff of 70% by the GOLD criteria [25].

**Figure 2:**
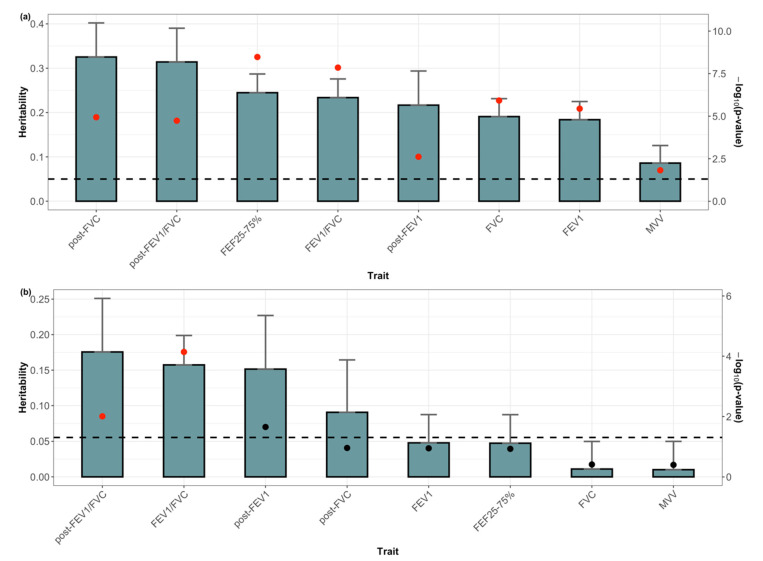
Single nucleotide polymorphism (SNP) heritability of eight lung function traits. SNP heritability of subject-specific means (**a**) and the annual change rates (**b**) in eight lung function traits. Error bars correspond to standard error. The black dots on the bars represent *p*-values. The black dashed line indicates the 0.05 significance level. Red dots indicate significant findings at a false-discovery rate of 0.05. FVC, forced vital capacity; FEV_1_, forced expiratory volume in 1 s; FEV_1_/FVC, forced expiratory volume in 1 s to forced vital capacity ratio; FEF 25–75%, the average forced expiratory flow during the mid (25–75%) portion of the FVC; MVV, maximal voluntary ventilation; post-FVC, post-bronchodilator test for FVC; post- FEV_1_, post-bronchodilator test for FEV_1_; post- FEV_1_/FVC, post-bronchodilator test for FEV_1_/ FVC ratio.

Both ${\hat{\beta }}_{0}$ and $\hat{\beta }$ were transformed using the rank-based inverse normal transformation and used to estimate $h_{0}^{2}$ and $h_{1}^{2}$. Figure 2a shows the estimates of heritability represented by $h_{0}^{2}$. All lung function traits were significant at the false-discovery rate (FDR)-adjusted at 0.05 significance level. Post-bronchodilator FVC exhibited the largest $h_{0}^{2}$ (0.325, *p* = 1.16 × 10^−5^) followed by post-bronchodilator FEV_1_/FVC (0.314, *p* = 1.86 × 10^−5^). Supplementary Figure S2 compares the cross-sectional SNP heritability at each period with $h_{0}^{2}$. The results showed that the mean cross-sectional SNP heritability at each period and $h_{0}^{2}$ did not differ markedly. For each lung function trait, $h_{1}^{2}$ was < $h_{0}^{2}$ (Figure 2b). Post-bronchodilator FEV_1_/FVC exhibited the highest $h_{1}^{2}$ (0.176, *p* = 0.0099) followed by FEV_1_/FVC (0.158 *p* = 4.91 × 10^−5^; Detailed estimation results are listed in Supplementary Tables S3 and S14). Supplementary Table S4 shows the results when the subject-specific mean was also included as a covariate; here, $\beta_{1}$ was used as a response variable. The results showed that $h_{1}^{2}$ remained significant for FEV_1_/FVC and post-bronchodilator FEV_1_/FVC even after adjusting for the baseline effect.

For lung function traits with significant $h_{1}^{2}$ (FEV_1_/FVC, and post-bronchodilator FEV_1_/FVC), we calculated the correlation ($\rho_{g}$) between genetic components for the subject-specific mean (${\hat{\beta }}_{0}$) and annual change rate (${\hat{\beta }}_{1}$). Supplementary Figure S3 shows the phenotypic correlation between subject-specific means and annual change rates of FEV_1_/FVC and post-bronchodilator FEV_1_/FVC; their correlations without any adjustments were 0.24 and 0.22, respectively. Table 2 shows $\rho_{g}$ and $\rho_{e}$. $\rho_{g}$ indicates a genetic correlation where the relative proportions are shared between subject-specific means and annual change rates. Since we wanted to check genetic components between ${\hat{\beta }}_{0}$ and ${\hat{\beta }}_{1}$ and their shared genetic effects, we did not adjust the baseline in the genetic correlation model. The results showed that ≥50% of genetic components were significantly shared between subject-specific means and annual change rates ($\rho_{g}$ = 0.628, *p* = 4.59 × 10^−5^ for FEV_1_/FVC; $\rho_{g}$ = 0.466, *p* = 0.022 for post-bronchodilator FEV_1_/FVC). Table 2 also shows the residual phenotypic correlations ($\rho_{e}$) between subject-specific means and annual change rates. The $\rho_{e}$ indicates the relative proportions of environmental variances shared between subject-specific means and annual change rates. Residual phenotypic correlations were much smaller than $\rho_{g}$ ($\rho_{e}$ = 0.117 for FEV_1_/FVC; $\rho_{e}$ = 0.155 for post-bronchodilator FEV_1_/FVC), which indicates that subject-specific means and annual change rates may be affected by different environmental factors. We also checked other traits but did not find any significant genetic correlations.

### 2.3. Effect of Smoking on Heritability Estimates of the Eight Lung Function Traits

**Figure 3:**
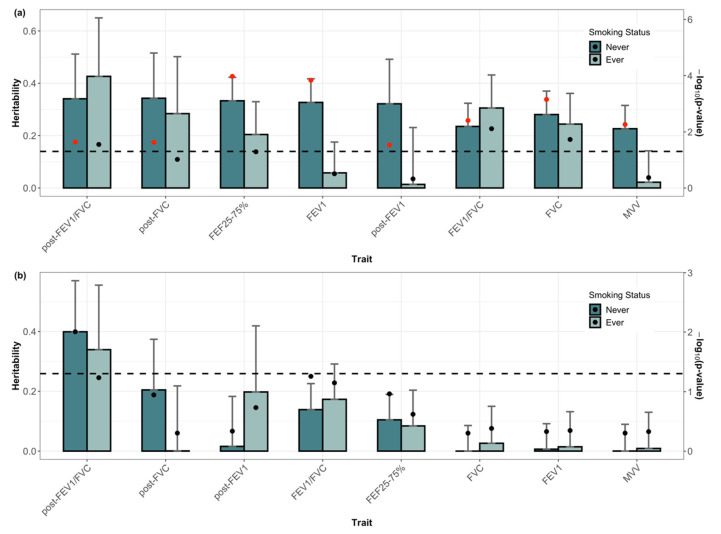
Single nucleotide polymorphism (SNP) heritability of eight lung function traits in never- and ever-smoker groups. SNP heritability of subject-specific means (**a**) and the annual change rates (**b**). Error bars correspond to standard error. The black dot on the bars represent *p*-values. The black dashed line indicates the 0.05 significance level. Red dots indicate significant findings at a false-discovery rate of 0.05. FVC, forced vital capacity; FEV_1_, forced expiratory volume in 1 s; FEV_1_/FVC, forced expiratory volume in 1 s to forced vital capacity ratio; FEF 25–75%, the average forced expiratory flow during the mid (25–75%) portion of the FVC; MVV, maximal voluntary ventilation; post-FVC, post-bronchodilator test for FVC; post-FEV_1_, post-bronchodilator test for FEV_1_; post- FEV_1_/FVC, post-bronchodilator test for FEV_1_/FVC ratio.

Subjects were separated into ever- and never-smokers and subgroup analyses were performed. Supplementary Table S5 shows the subject-specific means (${\hat{\beta }}_{0}$) and annual change rates (${\hat{\beta }}_{1}$) of subgroups. Significant differences between ever- and never-smokers were observed for all subject-specific means and annual change rates. For both groups, SNP heritability of subject-specific means ($h_{0}^{2}$) and SNP heritability of annual change rates ($h_{1}^{2}$) were separately estimated (Figure 3 and Supplementary Tables S6 and S7). The estimated $h_{0}^{2}$ in the never-smoker group was higher than that of ever-smokers, except for FEV_1_/FVC and post-bronchodilator FEV_1_/FVC. However, except for the post-bronchodilator FEV_1_/FVC of the never-smoker group, $h_{1}^{2}$ of the other traits was not significant at the 0.05 level.

We also evaluated the heritability of the SNP–smoking interaction ($h_{G\times S}^{2}$), which estimates the variances explained by the SNP and smoking interaction effect, for lung function traits with significant $h_{0}^{2}$ and $h_{1}^{2}$. All eight lung function traits exhibited significant $h_{0}^{2}$ values but none showed a significant $h_{G0\times S}^{2}$ (Supplementary Table S8). For the annual change rate, only post-bronchodilator FEV_1_/FVC showed significant estimation in $h_{1}^{2}$ and $h_{G1\times S}^{2}$. Table 3 shows that $h_{G1\times S}^{2}$ of post-bronchodilator FEV_1_/FVC was significant at the 0.05 significance level ($h_{G1\times S}^{2}$ = 0.402, *p* = 0.02). The other traits are listed in Supplementary Table S8. The significant results in the interaction analyses indicate the amount of genetic variance that would be affected by smoking.

### 2.4. Genome-Wide Association Studies of Subject-Specific Means and Annual Change Rates

To identify the disease susceptibility loci (DSL) of FEV_1_, FEV_1_/FVC, post-bronchodilator FEV_1_, and post-bronchodilator FEV_1_/FVC, we conducted GWAS with the subject-specific mean (${\hat{\beta }}_{0}$) and annual change rate (${\hat{\beta }}_{1}$) for each trait. Supplementary Tables S9 and S12 show the genome-wide significant SNPs at the significance level $\alpha =5\times 10^{-8}$ (for each significant trait, top 40 variants after clumping are listed in Supplementary Tables S10, S11 and S13). For subject-specific means, four DSL were identified. The genome-wide significant results of *CASC17* [26,27,28], *FAM13A* [12,26,29], *PID1* [30], and *TNXB* [31] have been previously reported. Genome-wide significance of rs62201158 for FEV_1_/FVC was reported for the first time. For annual change rates, rs2445936 was significantly associated with post-bronchodilator FEV_1_/FVC. Supplementary Figures S4 and S5 show the Manhattan and quantile–quantile plots of significant traits, which indicates that our GWAS preserves the nominal significance level. In addition, the inflation factors (lambda) were close to 1.

## 3. Discussion

In this study, we used two different heritability analyses of subject-specific means and annual change rates to estimate heritability for eight lung function traits. Using a 14-year follow-up of Korean population-based cohort dataset, we estimated the genetic correlations between the mean and annual change rate; subgroup and interaction analyses were also performed according to smoking status. To the best of our knowledge, this is the first study to report the heritability of the decline in lung function and other measurements in a population-based setting and Asian ancestry.

In our analysis, we found that the heritability of subject-specific means was significant for all eight lung function traits. The heritability of change in lung function, FEV_1_/FVC, and post-bronchodilator FEV_1_/FVC showed significant results. By comparing the estimated heritability via subgroup analyses between never- and ever-smokers, we found that the heritability of subject-specific means was higher in the never-smoker group. In the interaction analysis, we observed that the annual change in post-bronchodilator FEV_1_/FVC, FEV_1_/FVC, and MVV showed a significant or near-significant SNP–smoking interaction, thus allowing us to infer the amount of genetic variance that would be affected by smoking.

Previous cross-sectional estimates of pedigree-based heritability, compared with SNP-based heritability, have shown that lung function traits range from approximately 20 to 40% [9,17,32]. In the present study, the estimation of SNP heritability of subject-specific means of the lung function traits showed values similar to those in the previous studies. The overall estimated heritability for the eight traits ranged from approximately 9% (MVV) to 33% (post-bronchodilator FVC). The SNP heritability of annual change rates was lower than that of subject-specific means and ranged from approximately 1% (MVV) to 18% (post-bronchodilator FEV_1_/FVC), similar to previous studies on heritability decline [17]. This may indicate fewer genetic effects on the annual changes in the lung function than on the mean values. Interestingly, post-bronchodilator FEV_1_/FVC exhibited the most substantial estimated heritability for annual change rate among the eight traits; FEV_1_/FVC also displayed significant heritability. Moreover, the heritability of these traits was more than half of the subject-specific means, which suggested that genetic effects played an essential role in the annual change of these lung function traits.

Generally, the lung function of healthy subjects peaked in early adulthood, followed by a steady decline. Subjects who failed to reach the predicted level of peak lung function at an early age had a higher chance of being affected by COPD [33]. Recently, several studies showed that the decline in the FEV_1_ and FEV_1_/FVC patterns differ in subjects, with four different possible trajectories obtained using 29-year follow-up data [34]. These observations showed that the traditional notion of COPD being primarily caused by smoking was incomplete; besides, the peak level of lung function attained in youth constituted a major determinant of disease susceptibility [35,36]. It was also shown that genetic effects substantively contribute to these trajectories (up to 83%) [34]. Thus, longitudinal and perspective trajectory analysis is important to understand the mechanism of lung function.

Environmental factors could influence the estimation of lung function heritability. Traditionally, smoking is considered the most crucial factor to influence the decline in lung function [37]. However, up to 30% of patients with COPD worldwide never smoked [38]; therefore, exposure to other inhaled particles and gases can also lead to a decline in lung function [39]. In our study, we estimated SNP heritability for both subject-specific mean and annual change rates in never- and ever-smoker groups. We found that the two types of heritability analyses yielded different results, and that for some traits, different results were obtained between the two smoking groups. The heritability estimates of subject-specific means for FVC was higher (4%) for never- than ever-smokers. Moreover, the estimated heritability of FEV_1_/FVC and post-bronchodilator FEV_1_/FVC of ever-smokers was slightly, albeit significantly (at the 0.05 significance level) higher than that of never-smokers, with differences of 7% and 9%, respectively. Conversely, the heritability estimates of annual change rates did not reach significance, potentially owing to insufficient sample size. We also performed SNP–smoking interaction analysis, and annual change rates of post-bronchodilator FEV_1_/FVC, FEV_1_/FVC, and MVV showed significant and near-significant results, respectively, suggesting that the genetic variance for these three traits was affected by smoking in the middle-aged population.

Subject-specific means were strongly and positively genetically correlated (≥0.466), with annual change rates for FEV_1_/FVC and post-bronchodilator FEV_1_/FVC. As these metrics consist of genetic and environmental components, the positive correlations between their SNP effects indicate that subjects with higher genetic risk for subject-specific means of traits, such as FEV_1_/FVC and post-bronchodilator FEV_1_/FVC, tend to have a higher genetic risk for annual change rates as well. If the genetic effect for subject-specific means is fixed, the conditional variance of genetic effect on annual change rates ($\sigma_{g1}^{2}\left(1-\rho_{g}^{2}\right)$) becomes 0.0932 and 0.1364 for FEV_1_/FVC and post-bronchodilator FEV_1_/FVC, respectively, which indicates that the heritability of annual change rates is partially independent of the subject-specific mean genetic components. Therefore, we can conclude that a large proportion of the genetic effect on annual change rates is due to effects on subject-specific means. Our participants were aged 40 or older, with decreasing lung functions. Individuals with a higher lung function peak experience a lesser decline in lung function and our results can explain why the individuals with low peak lung function at an early age are often at a higher risk of developing disordered lung function, which is one of the parameters of lung function trajectories [34,40]. However, in view of different observations been reported, further studies are necessary to clarify this further.

We also performed genome-wide association analyses using subject-specific means and annual change rates of the lung function as the response, where sex, average age, and height were adjusted as covariates. Because of the small sample size, GWAS with the smoking subgroup were not conducted, and it is discussed in our future study as well as a validation study. The GWAS results for all samples, with a genome-wide significance level of 5 × 10^−8^, are shown in Supplementary Files (Supplementary Tables S9 and S12; Supplementary Figures S4 and S5). In the subject-specific means analysis, we detected that the association of rs4793538 with FEV_1_ was the most significant among the eight traits. This variant is located near *CASC17*, and interestingly, located in the upstream of *SOX9*, which was reported to be associated with FEV_1_ in previous studies [26,41]. *SOX9* was shown to be upregulated in adenocarcinoma of the lung, and its gene expression was associated with cell proliferation and lung development [42]. For the FEV1/FVC ratio, we discovered rs2704589 within *FAM13A*, which was found to be associated with FEV_1_/FVC ratio and COPD susceptibility in the prior GWAS [12,43,44,45]. A previous study with a different Korean population found that expression of *FAM13A* is much higher in the lung tissue of COPD cases compared to controls, and the increased gene expression levels are associated with the risk allele [45]. We also detected that rs62201158, located between *SPHKAP* and *PID1*, are associated with FEV_1_/FVC for the first time. In the prior investigations, *PID1* was associated with FEV_1_/FVC ratio [12] and was also found to be overexpressed in COPD lung tissues [45]. Previous studies have indicated that *PID1* has a role in tissue homeostasis and cell growth [46], and that overexpression of *PID1* induces mitochondrial dysfunction in adipocytes [47]. Furthermore, *PID1* overexpression may contribute to COPD development via mitochondrial malfunction and excess reactive oxygen species [45,48]. Another identified significant SNP, rs9391733, which mapped to *TNXB*, was also previously reported to be associated with lung function traits (FEV_1_ and FEV_1_/FVC) and COPD [41,49,50].

In the annual change rates analysis, we identified that SNP rs2445936 near *CEP164* was associated with post-bronchodilator FEV_1_/FVC. *CEP164* was previously reported to be critical for ciliogenesis during differentiation of airway multiciliated cells implicated in respiratory function [51]. In COPD, the ciliary structure and function are impaired, affecting the clearance of harmful inhaled debris and particles, such as tobacco smoke and fumes, leading to chronic inflammation and hyperinflation of the lung [52,53]. Therefore, the link between post-bronchodilator FEV_1_/FVC and the locus in *CEP164* in our analysis provides the evidence that the cilia in the airway play an important role in lung function decline.

One of the limitations of this study was sample size. Although the sample size was sufficiently large to support analysis of the complete data, it was limited for subgroup analysis. This led to significant standard errors for heritability estimation in the analysis. Previous studies have also reported that SNP heritability estimates are affected by the sample size when using GCTA [21]. Thus, to yield more reliable results, a larger sample size will be needed in future studies. In addition, the study population was based on a heterogenous community cohort, and smoking exposure was confounded with sex and height variables.

In summary, we estimated SNP heritability for eight lung function traits using a two-stage method to estimate cross-sectional averages and annual change rates in a Korean population-based longitudinal dataset. Our findings will help to understand aetiology of lung function decline and may facilitate the discovery of genetic factors influencing lung function-related traits. A larger sample size and novel statistical approaches will be required in future studies to validate and extend our findings.

## 4. Methods

### 4.1. Study Subjects

The KoGES cohort [23] consists of participants residing in Ansan (an urban area) and Ansung (a rural area) in the Gyeonggi Province of South Korea. KoGES was designed to investigate genetic, environmental, and behavioural risk factors of common complex diseases and causes of death in Koreans with long-term follow-up [54]. The baseline survey was completed in 2001–2002; 10,030 participants aged 40–69 years were recruited and followed for 14 years. Measurements were obtained from each participant every 2 years. To minimise potential bias and loss of power, we considered the data only from participants who were followed more than three times. Consequently, 6622 participants (3441 men and 3181 women; 3569 Ansan and 3053 Ansung) were identified, for whom both genotypic and phenotypic information were available. For the genotype data, 3,352,722 SNPs were considered in the analysis after quality control and imputation procedures. The details of the procedures are included in Supplementary Text S1. All participants provided written informed consent, and the ethics committee of the Korean Centre for Disease Control and institutional review boards of the Korea University Ansan Hospital approved the study (IRB no. 2020AS0356)

### 4.2. Lung Function

We considered pre-bronchodilator absolute values, which included FVC, FEV_1_, FEV_1_/FVC ratio, the average forced expiratory flow during the mid (25–75%) portion of the FVC (FEF 25–75%), and maximal voluntary ventilation (MVV). To investigate the exact COPD prevalence as well as bronchodilator response in a sub-population, we also performed a post-bronchodilator test for FVC, FEV_1_, and FEV_1_/FVC ratio, after short-acting bronchodilator treatment. These traits were measured by technicians using a portable spirometer (Vmax-2130, Sensor Medics, Yorba Linda, CA, USA) according to standardised protocols of the American Thoracic Society [55]. All participants performed the pre-bronchodilator spirometry test until at least three repeated measurements were completed; an acceptable measure was determined when the differences between the largest and the next largest FVC and FEV_1_ values were within 0.15 L [55]. Additionally, a post-bronchodilator test was performed in participants from Ansan-cohort only. Fenoterol hydrobromide (400 μg, 2001–2008) or salbutamol (400 μg, 2009 onwards) was administered and each test was repeated after a 15 to 30 min delay. Calibration and quality control of spirometric examinations were performed regularly based on the American Thoracic Society guidelines [55,56,57].

### 4.3. Epidemiological Concept of $h_{0}^{2}$ and $h_{1}^{2}$

We assumed that the trait of subject *i* at time point *j* was *y_ij_*, and *y_ij_* was a subject-specific function of the subject’s age, age_ij_. For our data, *j* was a number from 1 to 8. The subject-specific function was *f_i_* and $\varepsilon_{ij}$ was a measurement error. Then we assumed the following:

(1) $$y_{ij}=f_{i}\left({\mathrm{age}}_{ij}\right)+\varepsilon_{ij},\;\varepsilon_{ij}\sim N\left(0,\sigma_{m}^{2}\right)$$

Based on this definition, we estimated the subject-specific means (which indicate the mean of lung function traits across all visits) and annual change rates for the trait; two different SNP-based heritability parameters, $h_{0}^{2}$ and $h_{1}^{2}$, were also defined for both (see Supplementary Text S2).

If $h_{0}^{2}$ is high, then the overall genetic effect on the cross-sectional average tends to be high, and $h_{0}^{2}$ is equivalent to the SNP heritability estimated using genome-wide complex trait analysis (GCTA) [24]. The average annual change affected by genetic components is indicated by $h_{1}^{2}$ > 0. Supplementary Figure S1 illustrates the practical concept for $h_{0}^{2}$ and $h_{1}^{2}$.

### 4.4. Estimation of $h_{0}^{2}$ and $h_{1}^{2}$

A two-stage method was used to calculate $h_{0}^{2}$ and $h_{1}^{2}$ based on the method of Yang et al. [24]. First, we fitted a simple linear regression model for the subjects during the same visiting time, adjusting for age for each trait, as in Equation (2), which was transformed from Equation (1) (see Supplementary Text S2). Each participant was measured up to eight times and participants with at least three measurements were analysed. Since variances in lung function traits were heterogeneous at the eight different time points, the residual variances for each time point were calculated from linear regressions after adjusting for age, sex, and height for absolute values. The inverse of the residual variances for trait *k* and time point *j* was taken as *w_jk_,* and the weighted linear regression was performed for each subject *i* using the following equation:

(2) $$y_{ijk}=\beta_{ik0}+\beta_{ik1}\left(age_{ij}-{\bar{age}}_{i}\right)+\varepsilon_{ijk},\;\varepsilon_{ijk}\sim N\left(0,\frac{1}{w_{jk}}\sigma_{ik}^{2}\right)$$

Here, ${\bar{age}}_{i}$ indicates the mean age at the observed time points. $\beta_{ik0}$ indicates the subject-specific mean of subject *i* for trait *k* at ${\bar{age}}_{i}$, and $\beta_{ik1}$ indicates the annual change rate. Since we subtracted the mean age from each time point, the estimate of $\beta_{ik0}$ and $\beta_{ik1}$ is orthogonal. Since we considered several time points in the analysis, the subject-specific mean was more important than the baseline value. We then applied a rank-based inverse normal transformation to ${\hat{\beta }}_{ik0}$ and ${\hat{\beta }}_{ik1}$, and the bivariate linear mixed model was applied to estimate $h_{0}^{2}$ and $h_{1}^{2}$ after adjusting for sex, mean age, and mean height. The genetic relationship matrix (GRM) between pairs of individuals from the genome-wide variants were applied in estimation of variance covariance matrix in the linear mixed model (Supplementary Text S2), and the PC plot, based on GRM, was also generated (Supplementary Figure S6). This model was estimated using the “--reml-bivar” option of GCTA [58]. The genetic correlation between $\beta_{ik0}$ and $\beta_{ik1}$ was also generated using this model and option. If the subject-specific mean ($\beta_{ik0}$) is smaller, the positive genetic correlation indicates the change rate ($\beta_{ik1}$), which is a negative value, getting smaller, and vice versa.

### 4.5. Subgroup Analysis by Smoking Status

To conduct subgroup analyses, we separated subjects into ever- and never-smoker groups. The ever-smoking group included past and current smokers. The heritability of subject-specific means and annual change rates were estimated using GCTA for the two groups.

### 4.6. Estimating Heritability Attributed to SNP–Smoking Interaction

To estimate the variance in the SNP–smoking interaction for both subject-specific means and annual change rates, we applied the genotype-environment (GxE) interaction model, in which the main effects of environmental factors were included as fixed effects, while GxE interaction effects were treated as random effects (using the “--gxe” option of GCTA).

### 4.7. Genome-Wide Association Studies

Genome-wide association studies were conducted for eight lung function traits. Quality control and imputation were conducted, and the detailed procedure is described in Supplementary Text S1. Linear regression for $\beta_{ik0}$ and $\beta_{ik1}$ was conducted after adjusting for age, sex, height, and principle components (PCs, PC1 to PC10). The analyses were conducted using ONETOOL [59] and PLINK [60].

## Figures and Tables

**Table 1:** Descriptive characteristics of study participants with more than three follow-up visits.

Characteristic	ALL	Never Smoker	Ever Smoker	*p*-Value
Sample Size, n	6622	3009	2095	-
Female, n (%)	3181 (48.04%)	2588 (86%)	104 (5%)	<2.2 × 10^−16^
Age, year (means ± SD)	51.51 ± 8.52	51.19 ± 8.3	50.54 ± 7.94	0.005
Height, cm (means ± SD)	160.3 ± 8.57	155.9 ± 7.02	166.6 ± 6.26	<2.2 × 10^−16^

The “All” status included 5104 participants with a smoking history, and these participants were considered in the smoking subgroup analysis and SNP–smoking interaction analysis. Some participants had no smoking status. *p*-values were generated from statistical tests comparing never-smoker and ever-smoker groups. The Chi-square test was used for females and a *t*-test was performed for age and height. The sample sizes reported here are the number of samples used in the heritability estimation.

**Table 2:** Genetic correlation of subject-specific means (${\hat{\beta }}_{0}$ ) and annual change rates (${\hat{\beta }}_{1}$ ) in lung function traits with significant *p*-values (*p* < 0.05).

Trait	$\sigma_{g0}^{2}$	$\sigma_{g1}^{2}$	$\sigma_{g0}\sigma_{g1}\rho_{g}$	$\rho_{g}$	$s.e.(\rho_{g})$	$p-\mathrm{Value}\;(\rho_{g})$	$\rho_{e}$	$s.e.\;(\rho_{e})$
FEV_1_/FVC (%)	0.1807	0.1538	0.1047	0.6279	0.1466	0.0000459	0.117	0.0121
post-FEV_1_/FVC (%)	0.2593	0.1742	0.099	0.466	0.2156	0.0219	0.155	0.0165

$\sigma_{g0}^{2}$ = genetic variance of subject-specific mean; $\sigma_{g1}^{2}$ = genetic variance of annual change rates; $\sigma_{g0}\sigma_{g1}\rho_{g}$ = genetic covariance; $\rho_{g}$ = genetic correlation; $\rho_{e}$ = correlation of residuals; FEV_1_/FVC, forced expiratory volume in 1 s to forced vital capacity ratio; post-FEV_1_/FVC, post-bronchodilator test FEV_1_/FVC ratio.

**Table 3:** Heritability of SNP–smoking interaction for annual change rates with significant *p*-value (*p* < 0.05).

Traits	Subject-Specific Means			Annual Change Rates		
	$h_{gxe}^{2}$	s.e.	*p*-Value	$h_{gxe}^{2}$	s.e.	*p*-Value
post-FEV_1_/FVC (%)	0.206	0.191	1.37 × 10^−1^	0.402	0.199	2.12 × 10^−2^

SNP, single nucleotide polymorphism; $h_{gxe}^{2}$, heritability of SNP–smoking interaction; s.e., standard error; post-FEV_1_/FVC, post-bronchodilator test FEV_1_/FVC ratio.

## Data Availability

The information of the datasets analysed during the current study is available in the KoGES website [https://nih.go.kr/contents.es?mid=a50401010100] (accessed on 1 March 2019).

## Supplementary Materials

The following supporting information can be downloaded at: https://www.mdpi.com/article/10.3390/genes13071261/s1, Text S1: Genotyping, Quality Control (QC), and Imputation; Text S2: Supplementary methods; Figure S1: The illustrative example of $h_{0}^{2}$ and $h_{1}^{2}$; Figure S2: Cross-sectional heritability estimates; Figure S3: (A) Scatter plots between subject-specific mean (${\hat{\beta }}_{0}$) and annual change rate (${\hat{\beta }}_{1}$). (B) Scatter plots between annual change rate rates (${\hat{\beta }}_{1}$) and observed values; Figure S4: Manhattan Plot and Q-Q plot of subject-specific means (${\hat{\beta }}_{0}$) for (A) FEV_1_, (B) FEV_1_/FVC; Figure S5: Manhattan Plot and Q-Q plot of annual change rates (${\hat{\beta }}_{1}$) for (A) post- FEV_1_/FVC; Figure S6: Plot of principle component which based one genomic relationship matrix (GRM); Table S1: Sample numbers of the 8 lung function traits in each period; Table S2: Sample sizes in heritability estimation and means of subject-specific mean (${\hat{\beta }}_{0}$) and annual change rate (${\hat{\beta }}_{1}$) for 8 lung function traits; Table S3: SNP heritability of subject-specific means ($h_{0}^{2}$) and annual change rates ($h_{1}^{2}$) of lung function traits; Table S4: Effects of the subject-specific mean on heritability of annual change rate; Table S5: Summary of subject-specific means (${\hat{\beta }}_{0}$) and annual change rates (${\hat{\beta }}_{1}$) of 8 lung function traits in never-and ever-smoker groups; Table S6: SNP heritability of subject-specific means ($h_{0}^{2}$) and annual change rates ($h_{1}^{2}$) of lung function traits in never smoking group; Table S7: SNP heritability of subject-specific means ($h_{0}^{2}$) and annual change rates ($h_{1}^{2}$) of lung function traits in ever smoking group; Table S8: Heritability of SNP-by-smoking ($h_{G\times S}^{2}$) interaction for subject-specific means and annual change rates; Table S9: Genome-wide association analysis results of subject-specific means (${\hat{\beta }}_{0}$) for lung function; Table S10: Genome-wide association analysis results of subject-specific means (${\hat{\beta }}_{0}$) for FEV_1_. Top 40 variants are listed after clumping; Table S11: Genome-wide association analysis results of subject-specific means (${\hat{\beta }}_{0}$) for FEV_1_/FVC. Top 40 variants are listed after clumping; Table S12: Genome-wide association analysis results of annual change rate (${\hat{\beta }}_{1}$) for lung function; Table S13: Genome-wide association analysis results of annual change rate (${\hat{\beta }}_{1}$) for post-FEV_1_/FVC. Top 40 variants are listed after clumping; Table S14: The fixed effects estimations used in SNP the heritability estimation models of subject-specific means ($h_{0}^{2}$) and annual change rates ($h_{1}^{2}$). References [61,62,63,64,65] are cited in the Supplementary materials.

## Abbreviations

COPD	(Chronic obstructive pulmonary disease)
GWAS	(genome-wide association study)
FEV_1_	(forced expiratory volume in 1 s)
FVC	(forced vital capacity)
FEF	25–75% (average forced expiratory flow during the mid (25–75%) portion of the FVC)
MVV	(SNP (single nucleotide polymorphism)
KoGES	(Korean Genome and Epidemiology Study)
GCTA	(genome-wide complex trait analysis)
GxE	(genotype-environment interaction)
PC	(principle components)
FDR	(false-discovery rate)
DSL	(disease susceptibility loci)
