# Supplementary material for: Heritability Analyses Uncover Shared Genetic Effects of Lung Function and Change over Time
Source: Genes (Basel). 2022 Jul 15;13(7):1261. doi: 10.3390/genes13071261 (PMC9316642; doi:10.3390/genes13071261)
Supplement: Supplementary file 1 [file genes-13-01261-s001.zip › genes-1732014-supplementary.pdf]

Supplementary Material for "Heritability Analyses Uncover Shared Genetic Effects for Lung Function Levels and Change Over Time"

Donghe Li[1,9], Woo Jin Kim[2], Jahoon An[3], Soriul Kim[4], Seung Ku Lee[4], Ahra Do[1], Wonji Kim[5], Sanghun Lee[6], Dankyu Yoon[11], Kwangbae Lee[12], Seounguk Ha[12], Edwin K Silverman[5], Michael Cho[5], Chol Shin[4,7*], and Sungho Won[1,3,8,10*]

[1]Interdisciplinary Program in Bioinformatics, Seoul National University, Seoul, Republic of Korea;

[2]Department of Internal Medicine and Environmental Health Center, School of Medicine, Kangwon National University, Chuncheon, Republic of Korea;

[3]Department of Public Health Science, Graduate School of Public Health, Seoul National University, Seoul, Republic of Korea;

[4]Institute for Human Genomic Study, College of Medicine, Korea University, Seoul, Republic of Korea;

[5]Channing Division of Network Medicine and Division of Pulmonary and Critical Care Medicine, Brigham and Women's Hospital, Boston, Massachusetts;

[6]Department of Medical Consilience, Graduate School, Dankook University, Yongin, South Korea;

[7]Division of Pulmonary Sleep and Critical Care Medicine, Department of Internal Medicine, Korea University Ansan Hospital, Ansan, Republic of Korea;

[8]Institute of Health and Environment, Seoul National University, Seoul, Republic of Korea

[9]Department of Medicine (Biomedical Genetics), Boston University School of Medicine, Boston, MA, USA.

[10]RexSoft Inc., Seoul, Republic of Korea.

[11]Division of Allergy and Respiratory Disease Research, Department of Chronic Disease Convergence Research, National Institute of Health, Korea Disease Control and Prevention Agency, Cheongju, Republic of Korea.

[12]Korea Medical Institute, Seoul, Republic of Korea.

Donghe Li and Woo Jin Kim contributed equally to this work.

Correspondence and requests for reprints should be addressed to both Sungho Won and Chol Shin. Sungho Won, Department of Public Health Science, Seoul National University, 1 Gwanak-ro Gwanak-gu Seoul 08826, Korea. Tel: +82-2-880-2714, Fax: +82-303-0942-2714, E-mail: won1@snu.ac.kr

Chol Shin, Division of Pulmonary Sleep and Critical Care Medicine, Department of Internal Medicine, Korea University Ansan Hospital, 123, Jeokgeum-ro, Danwon-gu, Ansan, 15355, Republic of Korea. Tel: +82-31-412-5603, Fax: +82-31-412-5604, E-mail: chol-shin@korea.ac.kr

Supplementary Text S1. Genotyping, Quality Control (QC), and Imputation

All patients were genotyped using the Affymetrix Genome-Wide Human SNP array 5.01 (Santa Clara, CA, USA). For QC tests, we excluded SNPs with missing genotype call rates > 0.05, minor allele frequencies < 0.05, and Hardy-Weinberg equilibrium *P*-values < 10^{-5}. Additionally, participants with missing genotype call rates > 0.05 or sex inconsistencies were excluded. QC was performed using PLINK [1] and ONETOOL [2]. Following QC, 6,622 participants with 305,158 markers remained.

We conducted whole-genome imputation by using SHAPEIT2 [3] and IMPUTE2 [4, 5] for pre-phasing data and genotype imputation, respectively; the 1000 Genomes Phase 3 public dataset was used as a reference panel. To maintain imputation quality, we filtered out imputed SNPs with < 0.5 estimated imputation "info" score. The same QC procedure was also applied to these imputed SNPs, resulting in 6,622 participants with 3,352,722 SNPs being analyzed for SNP heritability estimation.

Supplementary Text S2

We assumed that the observed trait of subject i at time point j was y_{ij} and then we could assume that y_{ij} was expressed by its subject-specific function f_i of his and her age (age_{ij}) as follows:

$$y_{ij} = f_i(\text{age}_{ij}) + \varepsilon_{ij},\ \varepsilon_{ij} \sim N(0, \sigma_m^2).$$

By Taylor expansion, for subject i, the subject-specific function, f_i, became

$$f_i(\text{age}_{ij}) \approx f_i(\overline{\text{age}}_i) + f_i'(\overline{\text{age}}_i)(\text{age}_{ij} - \overline{\text{age}}_i) = \beta_{0i} + \beta_{1i}(\text{age}_{ij} - \overline{\text{age}}_i).$$

$$\beta_{0i} = \alpha_0^0 + \alpha_1^0 \text{sex}_i + \alpha_2^0 \overline{\text{age}}_i + \alpha_3^0 \overline{\text{height}}_i + g_i^0 + e_i^0,$$

$$\beta_{1i} = \alpha_0^1 + \alpha_1^1 \text{sex}_i + \alpha_2^1 \overline{\text{age}}_i + \alpha_3^1 \overline{\text{height}}_i + g_i^1 + e_i^1$$

$$\begin{pmatrix} g_i^0 \\ g_i^1 \end{pmatrix} \sim MVN\left(\begin{pmatrix} 0 \\ 0 \end{pmatrix}, \begin{pmatrix} \sigma_{g0}^2 & \sigma_{g0}\sigma_{g1}\rho_g \\ \sigma_{g0}\sigma_{g1}\rho & \sigma_{g1}^2 \end{pmatrix}\right), \begin{pmatrix} e_i^0 \\ e_i^1 \end{pmatrix} \sim MVN\left(\begin{pmatrix} 0 \\ 0 \end{pmatrix}, \begin{pmatrix} \sigma_{e0}^2 & \sigma_{e0}\sigma_{e1}\rho_e \\ \sigma_{e0}\sigma_{e1}\rho_e & \sigma_{e1}^2 \end{pmatrix}\right).$$

We defined the column vectors as follows:

$$\boldsymbol{\beta^0} = (\beta_{01} \quad \cdots \quad \beta_{0n})^t, \boldsymbol{\beta^1} = (\beta_{01} \quad \cdots \quad \beta_{0n})^t,$$

$$\boldsymbol{g^0} = (g_1^0 \quad \cdots \quad g_n^0)^t, \boldsymbol{g^1} = (g_1^1 \quad \cdots \quad g_n^1)^t$$

$$\boldsymbol{e^0} = (e_1^0 \quad \cdots \quad e_n^0)^t, \text{ and } \boldsymbol{e^1} = (e_1^1 \quad \cdots \quad e_n^1)^t.$$

Then based on the Lee et al. and Yang et al. paper[6, 7], we can assume that

$$\begin{pmatrix} \boldsymbol{\beta^0} \\ \boldsymbol{\beta^1} \end{pmatrix} = \begin{pmatrix} \alpha_0^0 \\ \alpha_0^1 \end{pmatrix} \otimes \mathbf{1} + \begin{pmatrix} \alpha_1^0 \\ \alpha_1^1 \end{pmatrix} \otimes \mathbf{sex} + \begin{pmatrix} \alpha_2^0 \\ \alpha_2^1 \end{pmatrix} \otimes \overline{\mathbf{age}} + \begin{pmatrix} \alpha_3^0 \\ \alpha_3^1 \end{pmatrix} \otimes \overline{\mathbf{height}} + \begin{pmatrix} \boldsymbol{g^0} \\ \boldsymbol{g^1} \end{pmatrix} + \begin{pmatrix} \boldsymbol{e^0} \\ \boldsymbol{e^1} \end{pmatrix},$$

$$\begin{pmatrix} \boldsymbol{g^0} \\ \boldsymbol{g^1} \end{pmatrix} \sim MVN\left(\begin{pmatrix} 0 \\ 0 \end{pmatrix} \otimes \mathbf{1}, \begin{pmatrix} \sigma_{g0}^2 & \sigma_{g0}\sigma_{g1}\rho_g \\ \sigma_{g0}\sigma_{g1}\rho & \sigma_{g1}^2 \end{pmatrix} \otimes \mathbf{GRM}\right),$$

$$\begin{pmatrix} \boldsymbol{e^0} \\ \boldsymbol{e^1} \end{pmatrix} \sim MVN\left(\begin{pmatrix} 0 \\ 0 \end{pmatrix} \otimes \mathbf{1}, \begin{pmatrix} \sigma_{e0}^2 & \sigma_{e0}\sigma_{e1}\rho_e \\ \sigma_{e0}\sigma_{e1}\rho_e & \sigma_{e1}^2 \end{pmatrix} \otimes \mathbf{I}\right), \ldots (1).$$

Here, GRM indicates the genetic relationship matrix between pairs of individuals from the genome-wide variants. Based on this definition, we defined the following two different heritabilities.

$$h_0^2 = \frac{\sigma_{g0}^2}{\sigma_{g0}^2 + \sigma_0^2},\ h_1^2 = \frac{\sigma_{g1}^2}{\sigma_{g1}^2 + \sigma_1^2}.$$

ρ_g indicates the correlations between genetic components for β_{0i} and β_{1i}.

Supplementary Table S1. Sample numbers of the 8 lung function traits in each period.

Triats	P1	P2	P3	P4	P5	P6	P7	P8
FVC (L)	6622	4832	5923	5287	5127	3949	4532	4477
FEV_1 (L)	6622	4833	5922	5287	5127	3946	4532	4476
FEV_1/FVC (%)	6622	4833	5923	5286	5128	3949	4532	4477
FEF25-75% (L/sec)	6622	4833	5922	5282	5128	3948	4532	4477
MVV (L/min)	6614	4829	5913	5279	5127	3946	4530	4476
post-FVC (L)	3489	3275	3058	2759	2683	2469	2270	2107
post-FEV_1 (L)	3490	3275	3058	2759	2682	2469	2270	2107
post-FEV_1/FVC (%)	3490	3275	3057	2759	2684	2469	2270	2107

P stands for period (time point). 8 periods were obseved in the data set. Sample size here is the number of samples used in heritability estimation. FVC, forced vital capacity; FEV_1, forced expiratory volume in one second; FEF 25–75, average forced expiratory flow during the mid (25–75%) portion of the FVC; MVV maximal voluntary ventilation; post-FVC, post-bronchodilator test for FVC; post- FEV_1, post-bronchodilator test for FEV_1; post- FEV_1/FVC, post-bronchodilator test for FEV_1/ FVC ratio.

Supplementary Table S2. Sample sizes in heritability estimation and means of subject-specific mean ($\hat{\beta}_0$) and annual change rate ($\hat{\beta}_1$) for 8 lung function traits.

Traits	Sample size	Subject-specific mean ($\hat{\beta}_0$) Mean (SD)	Annual change rate ($\hat{\beta}_1$) Mean (SD)
FVC (L)	6622	3.467 (0.832)	−0.036 (0.033)
FEV_1 (L)	6622	2.695 (0.649)	−0.04 (0.026)
FEV_1/FVC (%)	6622	77.977 (6.802)	−0.338 (0.512)
$FEF_{25–75}$ (L/sec)	6622	2.604 (0.958)	−0.074 (0.06)
MVV (L/min)	6614	103.775 (29.545)	−2.266 (1.997)
post-FVC (L)	3489	3.62 (0.808)	−0.037 (0.025)
post-FEV_1 (L)	3490	2.932 (0.635)	−0.038 (0.021)
post-FEV_1FVC (%)	3490	81.331 (5.74)	−0.214 (0.407)

FVC, forced vital capacity; FEV_1, forced expiratory volume in one second; FEF 25–75, average forced expiratory flow during the mid (25–75%) portion of the FVC; MVV maximal voluntary ventilation; post-FVC, post-bronchodilator test for FVC; post- FEV_1, post-bronchodilator test for FEV_1; post- FEV_1/FVC, post-bronchodilator test for FEV_1/ FVC ratio.

Supplementary Table S3. SNP heritability of subject-specific means (h_0^2) and annual change rates (h_1^2) of lung function traits.

Traits	Subject-specific means				Annual change rates			
	h_0^2	s.e	P-value	FDR	h_1^2	s.e	P-value	FDR
FVC (L)	0.191	0.040	1.21E-06	2.42E-06	0.011	0.039	3.88E-01	4.00E-01
FEV_1 (L)	0.184	0.041	3.65E-06	6.25E-06	0.048	0.039	1.12E-01	1.57E-01
FEV_1/FVC (%)	0.234	0.042	1.40E-08	4.21E-08	0.157	0.041	7.25E-05	8.70E-04
FEF25-75% (L/sec)	0.245	0.042	3.34E-09	2.00E-08	0.047	0.040	1.17E-01	1.57E-01
MVV (L/min)	0.086	0.040	1.52E-02	1.52E-02	0.010	0.040	4.00E-01	4.00E-01
post-FVC (L)	0.325	0.077	1.16E-05	1.74E-05	0.091	0.074	1.10E-01	1.57E-01
post-FEV_1 (L)	0.217	0.077	2.45E-03	2.94E-03	0.151	0.075	2.24E-02	5.38E-02
post-FEV_1/FVC (%)	0.314	0.076	1.86E-05	2.47E-05	0.176	0.075	9.92E-03	3.97E-02

h_0^2, SNP heritability of subject-specific means; h_1^2, SNP heritability of annual change rates; **s.e,** standard error; FDR, false discovery rate. FVC, forced vital capacity; FEV_1, forced expiratory volume in one second; FEF 25–75, average forced expiratory flow during the mid (25–75%) portion of the FVC; MVV maximal voluntary ventilation; post-FVC, post-bronchodilator test for FVC; post- FEV_1, post-bronchodilator test for FEV_1; post- FEV_1/FVC, post-bronchodilator test for FEV_1/ FVC ratio.

Supplementary Table S4. Effects of the subject-specific mean on heritability of annual change rate.

Traits	Covariates	h_1^2	s.e	P-value
FEV_1/FVC (%)	Age, sex, height	0.158	0.042	4.91×10^{-5}
	Age, sex, height β_{0i}	0.123	0.042	1.13×10^{-3}
post- FEV_1/FVC (%)	Age, sex, height	0.183	0.075	6.18×10^{-3}
	Age, sex, height β_{0i}	0.152	0.076	2.01×10^{-2}

We estimated h_1^2 after adjusting the effects of age, sex and subject-specific mean (β_{0i}), and it was compared with its estimates without adjusting the effect of the subject-specific mean (β_{0i}). FEV_1/FVC, forced expiratory volume in 1 sec to forced vital capacity ratio; post- FEV_1/FVC, post-bronchodilator test for FEV_1/ FVC ratio.

Supplementary Table S5. Summary of subject-specific means ($\hat{\beta}_0$) and annual change rates ($\hat{\beta}_1$) of 8 lung function traits in never-and ever-smoker groups.

Traits	Never-smoker			Ever-smoker			P-value	
	Sample Size	Means of subject-specific means (sd)	Means of annual change rates (sd)	Sample Size	Means of subject-specific means (sd)	Means of annual change rates (sd)	Subject-specific means	Annual change rates
FVC (L)	3008	3.045 (0.642)	-0.034 (0.023)	2095	4.051 (0.661)	-0.039 (0.027)	0	1.41e-09
FEV_1 (L)	3008	2.430 (0.520)	-0.036 (0.019)	2095	3.066 (0.592)	-0.046 (0.022)	1.27e-291	2.28e-57
FEV_1/FVC (%)	3008	79.815 (5.449)	-0.300 (0.430)	2095	75.540 (7.298)	-0.418 (0.406)	2.78e-107	3.65e-23
FEF25-75% (L/sec)	3009	2.526 (0.830)	-0.068 (0.047)	2095	2.712 (1.071)	-0.088 (0.051)	2.59e-11	7.10e-46
MVV (L/min)	3004	92.488 (24.048)	-2.052 (1.340)	2095	119.465 (27.968)	-2.583 (1.707)	6.62e-245	4.84e-32
post-FVC (L)	1524	3.181 (0.626)	-0.035 (0.018)	1182	4.144 (0.604)	-0.038 (0.023)	3.16e-278	1.11e-05
post-FEV_1 (L)	1525	2.628 (0.510)	-0.036 (0.014)	1182	3.296 (0.517)	-0.043 (0.018)	3.50e-207	3.31e-26
post-FEV_1FVC (%)	1525	82.772 (4.862)	-0.236 (0.312)	1182	79.667 (5.949)	-0.291 (0.331)	5.39e-46	1.01e-05

P-values were generated from statistical tests comparing never-smoker and ever-smoker groups. FVC, forced vital capacity; FEV_1, forced expiratory volume in one second; FEF 25–75, average forced expiratory flow during the mid (25–75%) portion of the FVC; MVV maximal voluntary ventilation; post-FVC, post-bronchodilator test for FVC; post- FEV_1, post-bronchodilator test for FEV_1; post- FEV_1/FVC, post-bronchodilator test for FEV_1/ FVC ratio.

Supplementary Table S6. SNP heritability of subject-specific means (h_0^2) and annual change rates (h_1^2) of lung function traits in never smoking group.

Traits	Subject-specific means				Annual change rates			
	h_0^2	s.e	P-value	FDR	h_1^2	s.e	P-value	FDR
FVC (L)	0.281	0.090	7.02E-04	2.10E-03	0.000	0.085	5.00E-01	5.00E-01
FEV_1 (L)	0.327	0.090	1.46E-04	6.01E-04	0.006	0.085	4.70E-01	5.00E-01
FEV_1/FVC (%)	0.235	0.089	3.96E-03	7.92E-03	0.139	0.087	5.55E-02	2.73E-01
FEF25-75% (L/sec)	0.333	0.090	1.06E-04	6.01E-04	0.105	0.085	1.09E-01	2.73E-01
MVV (L/min)	0.226	0.089	5.48E-03	9.36E-03	0.000	0.089	4.99E-01	5.00E-01
post-FVC (L)	0.343	0.173	2.36E-02	2.57E-02	0.204	0.169	1.14E-01	2.73E-01
post-FEV_1 (L)	0.322	0.170	2.94E-02	2.94E-02	0.016	0.167	4.63E-01	5.00E-01
post-FEV_1/FVC (%)	0.341	0.171	2.31E-02	2.57E-02	0.399	0.171	9.87E-03	1.18E-01

h_0^2, SNP heritability of subject-specific means; h_1^2, SNP heritability of annual change rates; **s.e,** standard error; FDR, false discovery rate. FVC, forced vital capacity; FEV_1, forced expiratory volume in one second; FEF 25–75, average forced expiratory flow during the mid (25–75%) portion of the FVC; MVV maximal voluntary ventilation; post-FVC, post-bronchodilator test for FVC; post- FEV_1, post-bronchodilator test for FEV_1; post- FEV_1/FVC, post-bronchodilator test for FEV_1/ FVC ratio.

Supplementary Table S7. SNP heritability of subject-specific means (h_0^2) and annual change rates (h_1^2) of lung function traits in ever smoking group.

Traits	Subject-specific means				Annual change rates			
	h_0^2	s.e	P-value	FDR	h_1^2	s.e	P-value	FDR
FVC (L)	0.244	0.117	1.87E-02	8.46E-02	0.026	0.124	4.16E-01	5.00E-01
FEV_1 (L)	0.058	0.118	3.12E-01	4.16E-01	0.014	0.117	4.51E-01	5.00E-01
FEV_1/FVC (%)	0.306	0.126	7.77E-03	8.46E-02	0.173	0.118	7.14E-02	3.66E-01
FEF25-75% (L/sec)	0.204	0.125	5.15E-02	1.03E-01	0.084	0.120	2.41E-01	5.00E-01
MVV (L/min)	0.022	0.120	4.28E-01	4.75E-01	0.009	0.121	4.71E-01	5.00E-01
post-FVC (L)	0.284	0.218	9.62E-02	1.65E-01	0.001	0.217	4.99E-01	5.00E-01
post-FEV_1 (L)	0.014	0.217	4.75E-01	4.75E-01	0.198	0.221	1.86E-01	5.00E-01
post-FEV_1/FVC (%)	0.426	0.224	2.82E-02	8.46E-02	0.339	0.216	5.85E-02	3.66E-01

h_0^2, SNP heritability of subject-specific means; h_1^2, SNP heritability of annual change rates; **s.e,** standard error; FDR, false discovery rate. FVC, forced vital capacity; FEV_1, forced expiratory volume in one second; FEF 25–75, average forced expiratory flow during the mid (25–75%) portion of the FVC; MVV maximal voluntary ventilation; post-FVC, post-bronchodilator test for FVC; post- FEV_1, post-bronchodilator test for FEV_1; post- FEV_1/FVC, post-bronchodilator test for FEV_1/ FVC ratio.

Supplementary Table S8. Heritability of SNP-by-smoking ($h^2_{G\times S}$) interaction for subject-specific means and annual change rates.

Traits	Sample Size	Subject-specific means			Annual change rates		
		$h^2_{G0\times S}$	s.e.	P-val	$h^2_{G1\times S}$	s.e.	P-val
FVC (L)	5103	0.052	0.098	0.29	-0.025	0.101	0.410
FEV_1 (L)	5103	-0.007	0.098	0.47	-0.090	0.091	0.162
FEV_1/FVC (%)	5103	0.079	0.101	0.22	0.138	0.101	0.079
FEF25-75% (L/sec)	5104	0.097	0.102	0.18	0.117	0.099	0.109
MVV (L/min)	5099	0.003	0.099	0.49	-0.150	0.094	0.072
post-FVC (L)	2706	-0.033	0.182	0.43	-0.089	0.189	0.328
post-FEV_1 (L)	2707	-0.074	0.186	0.35	-0.048	0.180	0.395
post-FEV_1/FVC (%)	2707	0.206	0.191	0.14	0.402	0.199	0.021

$h^2_{G\times S}$, heritability of SNP-smoking interaction. Here we dectected several negative values, these happened when there were limited (small) sample sizes or true heritability was too small to see the negative estimates. FVC, forced vital capacity; FEV_1, forced expiratory volume in one second; FEF 25–75, average forced expiratory flow during the mid (25–75%) portion of the FVC; MVV maximal voluntary ventilation; post-FVC, post-bronchodilator test for FVC; post- FEV_1, post-bronchodilator test for FEV_1; post- FEV_1/FVC, post-bronchodilator test for FEV_1/ FVC ratio.

Supplementary Table S9. Genome-wide association analysis results of subject-specific means ($\widehat{\beta}_0$) for lung function.

Trait	SNP	CHR	BP	A1	A2	MAF	HWE_P	REGION	GENE	BETA	P
FEV_1 (L)	rs4793538	17	69215928	T	C	0.3812	0.766	intergenic	CASC17(dist=17608),LOC102723505(dist=802064)	-0.06552	2.27E-10
FEV_1/FVC (%)	rs2704589	4	89848583	T	C	0.4707	0.9657	intronic	FAM13A	0.09519	5.16E-10
FEV_1/FVC (%)	rs62201158	2	229552897	G	A	0.128	0.1737	intergenic	SPHKAP(dist=506536),PID1(dist=335792)	0.1312	2.06E-08
FEV_1/FVC (%)	rs9391733	6	32059674	G	C	0.1638	0.5817	intronic	TNXB	0.1213	3.24E-08

Only the variants at genome-wide significant level of 5.0E-08 are listed.

Supplementary Table S10. Genome-wide association analysis results of subject-specific means ($\widehat{\beta}_0$) for FEV$_1$. Top 40 variants are listed after clumping.

SNP	CHR	BP	A1	A2	MAF	HWE_P	REGION	GENE	BETA	P
**rs4793538**	17	69215928	T	C	0.3812	0.766	intergenic	CASC17(dist=17608),LOC102723505(dist=802064)	-0.06552	2.27E-10
**rs16947654**	15	93799753	C	T	0.07745	0.2579	intergenic	RGMA(dist=167310),LOC101927153(dist=600036)	-0.09976	7.40E-08
**rs7033617**	9	118287508	A	G	0.2711	0.02447	intergenic	DEC1(dist=122585),LOC101928775(dist=214441)	0.05437	8.76E-07
**rs9651572**	11	15470776	G	A	0.4552	0.1522	intergenic	INSC(dist=202020),LOC102724957(dist=194655)	0.04849	1.15E-06
**SNP_A-4278378**	17	69111098	G	A	0.4111	0.2514	ncRNA_intronic	CASC17	0.04589	4.42E-06
**rs6856223**	4	185810329	C	T	0.2107	0.2833	intergenic	MIR3945HG(dist=33523),LINC01093(dist=3825)	-0.05576	5.34E-06
**rs2422362**	11	36758133	T	G	0.2434	0.1862	intergenic	C11orf74(dist=77292),LOC103312105(dist=1881681)	0.0536	5.89E-06
**rs3113720**	4	110030786	C	T	0.4071	0.5946	intronic	COL25A1	0.04543	7.02E-06
**rs9309966**	3	68159033	C	T	0.1607	0.5469	intronic	FAM19A1	0.05868	1.48E-05
**rs11086035**	19	9546460	T	C	0.3533	0.7065	upstream	ZNF266	-0.04537	1.51E-05
**rs6507116**	18	32632746	G	A	0.2559	0.4389	intronic	MAPRE2	0.04974	1.68E-05
**rs8104232**	19	42272611	G	A	0.4664	0.2185	intronic	CEACAM6	0.04211	1.94E-05
**SNP_A-1877081**	14	58140574	C	T	0.4085	0.757	intronic	SLC35F4	-0.0433	1.96E-05
**SNP_A-2105011**	3	30268517	A	G	0.0621	0.1982	intergenic	RBMS3(dist=216631),TGFBR2(dist=379477)	0.08602	2.13E-05
**rs79332079**	9	117816282	C	T	0.1108	0.385	intronic	TNC	-0.06597	2.36E-05
**SNP_A-2189286**	1	162673571	T	C	0.1351	0.4107	intronic	DDR2	-0.06099	2.42E-05
**rs7764368**	6	167094118	T	C	0.1631	0.7508	intronic	RPS6KA2	0.05726	2.49E-05
**rs11023551**	11	15389788	T	C	0.3241	0.4499	intergenic	INSC(dist=121032),LOC102724957(dist=275643)	-0.04448	2.60E-05
**rs6478176**	9	118258940	G	A	0.1891	0.0883	intergenic	DEC1(dist=94017),LOC101928775(dist=243009)	0.05362	2.65E-05
**rs6501837**	17	73822415	A	G	0.3649	0.4231	downstream	UNC13D,UNK	0.04425	2.74E-05
**SNP_A-2009412**	3	187912292	G	T	0.4301	0.8446	intronic	LPP	0.04182	2.92E-05
**SNP_A-2133065**	15	51653014	T	C	0.198	0.7622	intronic	GLDN	0.05189	3.00E-05
**rs32473**	5	171176059	A	G	0.151	0.2376	intergenic	FGF18(dist=291429),SMIM23(dist=36817)	0.05764	3.44E-05
**rs78451696**	18	64442995	C	T	0.07739	0.2235	intergenic	CDH19(dist=171620),MIR5011(dist=305826)	-0.07702	3.79E-05
**rs11944979**	4	89634548	T	C	0.2926	0.4365	ncRNA_intronic	FAM13A-AS1	-0.04504	3.87E-05
**rs2250660**	2	233752551	C	G	0.2939	0.3581	intronic	NGEF	-0.04562	3.96E-05
**rs10953347**	7	101160036	T	G	0.1084	0.241	intronic	COL26A1	-0.06652	4.20E-05
**rs61823898**	1	222152268	T	C	0.142	1	intergenic	LOC101929771(dist=138260),HHIPL2(dist=543334)	0.0589	4.28E-05
**rs77090961**	6	55682792	G	C	0.1002	0.3075	intronic	BMP5	-0.06725	4.56E-05
**rs1841673**	8	70066120	A	G	0.255	0.1344	intergenic	LINC01592(dist=49695),LINC01603(dist=270986)	-0.04679	4.75E-05
**rs9987404**	8	41758766	T	C	0.2519	0.3138	intergenic	ANK1(dist=4486),KAT6A(dist=28231)	0.04683	4.81E-05
**SNP_A-2072696**	11	133100233	C	T	0.4523	0.5461	intronic	OPCML	-0.04069	4.84E-05
**rs320722**	7	137025827	C	G	0.4602	0.4242	intronic	PTN	0.04076	5.08E-05
**rs72956730**	11	88389320	T	C	0.06384	0.08418	intronic	GRM5	-0.08361	5.50E-05
**rs10180537**	2	37034503	G	A	0.1435	0.7259	intronic	VIT	-0.05679	5.95E-05
**rs140352854**	11	93923633	G	T	0.1583	0.4673	intergenic	PANX1(dist=8496),IZUMO1R(dist=115170)	-0.05505	6.03E-05
**SNP_A-2118316**	4	183479255	C	T	0.1687	0.6478	intronic	TENM3	-0.05313	6.05E-05
**rs142584564**	5	121384647	C	A	0.06793	0.1043	intergenic	SRFBP1(dist=20352),LOX(dist=14243)	-0.07889	6.64E-05
**rs6541823**	2	123120079	T	C	0.1322	0.6742	intergenic	TSN(dist=594651),CNTNAP5(dist=1662785)	0.05851	6.75E-05
**rs926309**	6	15463139	T	G	0.2025	0.5078	intronic	JARID2	-0.04938	6.86E-05

Supplementary Table S11. Genome-wide association analysis results of subject-specific means ($\widehat{\beta}_0$) for FEV_1/FVC. Top 40 variants are listed after clumping.

SNP	CHR	BP	A1	A2	MAF	HWE_P	REGION	GENE	BETA	P
**rs2704589**	4	89848583	T	C	0.4707	0.9657	intronic	FAM13A	0.09519	5.16E-10
**rs62201158**	2	229552897	G	A	0.128	0.1737	intergenic	SPHKAP(dist=506536),PID1(dist=335792)	0.1312	2.06E-08
**rs9391733**	6	32059674	G	C	0.1638	0.5817	intronic	TNXB	0.1213	3.24E-08
**rs28383323**	6	32594039	A	G	0.2017	0.144	intergenic	HLA-DRB1(dist=36426),HLA-DQA1(dist=11144)	0.1534	1.24E-07
**SNP_A-4258568**	4	89912865	G	A	0.4074	0.8423	intronic	FAM13A	-0.07996	2.88E-07
**rs117982829**	6	32133380	G	C	0.05315	0.9133	ncRNA_intronic	PPT2-EGFL8	0.1824	4.12E-07
**rs8192575**	6	32166384	G	C	0.1488	0.7323	intronic	NOTCH4	0.1138	4.86E-07
**SNP_A-2128759**	16	77699400	A	G	0.3227	0.3792	intergenic	ADAMTS18(dist=230389),NUDT7(dist=56989)	-0.07634	3.14E-06
**rs1718313**	12	103175726	T	C	0.4288	0.1699	intergenic	IGF1(dist=301303),LINC00485(dist=27335)	-0.07372	3.15E-06
**rs2050911**	1	82384407	G	A	0.1536	0.3448	intronic	ADGRL2	0.09622	5.65E-06
**rs6773308**	3	27299577	G	A	0.1797	0.2151	intronic	NEK10	-0.09162	6.16E-06
**rs7448948**	5	172645164	C	T	0.08754	0.315	intergenic	BNIP1(dist=53774),NKX2-5(dist=13943)	0.1228	6.31E-06
**rs707928**	6	31742590	G	A	0.4389	0.4827	intronic	VWA7	-0.07794	7.01E-06
**rs11771139**	7	99736059	A	G	0.4066	0.7856	intergenic	MBLAC1(dist=9938),LAMTOR4(dist=10471)	-0.07121	7.97E-06
**rs9469051**	6	31741490	C	G	0.2763	0.5028	intronic	VWA7	-0.08515	8.54E-06
**rs1382167**	3	21636543	C	A	0.2232	0.707	intronic	ZNF385D	-0.08235	8.57E-06
**rs204883**	6	32032743	A	G	0.4689	0.1456	exonic	TNXB	0.07467	9.02E-06
**rs76598389**	17	76312757	T	C	0.1462	0.09554	intergenic	LOC100996291(dist=38184),SOCS3(dist=40101)	-0.1002	9.55E-06
**rs7893687**	10	104457603	A	T	0.3894	0.233	intronic	ARL3	-0.0686	1.11E-05
**rs9371349**	6	155044633	C	T	0.09709	1	intergenic	CNKSR3(dist=212880),SCAF8(dist=9879)	0.1136	1.32E-05
**SNP_A-1789562**	6	6693320	G	A	0.4877	0.5354	intergenic	LY86(dist=38104),RREB1(dist=414510)	0.06554	1.89E-05
**rs3735242**	7	99771849	A	G	0.3252	1	intronic	GPC2	0.07067	1.96E-05
**rs16947654**	15	93799753	C	T	0.07745	0.2579	intergenic	RGMA(dist=167310),LOC101927153(dist=600036)	-0.1214	2.07E-05
**rs76236233**	1	86037202	G	C	0.05498	0.1731	intronic	DDAH1	0.1443	2.10E-05
**rs74610824**	4	89760151	C	T	0.3042	0.2482	intronic	FAM13A	-0.07281	2.17E-05
**rs201082719**	6	26321792	A	C	0.07598	0.2424	intergenic	HIST1H4H(dist=36065),BTN3A2(dist=43595)	0.1535	2.19E-05
**rs1863492**	15	88656926	G	T	0.09705	0.6666	intronic	NTRK3	-0.1081	2.86E-05
**rs10754181**	1	196163178	A	T	0.4895	0.5782	intergenic	NONE(dist=NONE),KCNT2(dist=31732)	-0.06365	3.08E-05
**rs2833856**	21	33877691	C	T	0.2127	0.4767	intronic	EVA1C	0.07957	3.24E-05
**rs9403381**	6	142661156	T	G	0.3073	0.8792	intronic	ADGRG6	0.06974	3.31E-05
**rs2961291**	7	22681504	T	C	0.05761	0.4904	intergenic	LOC100506178(dist=67887),LOC401312(dist=8075)	-0.138	3.37E-05
**rs117689957**	6	32035861	G	A	0.05648	0.415	intronic	TNXB	0.145	3.42E-05
**rs12939972**	17	80007018	C	T	0.3417	0.5115	intronic	RFNG	0.0681	3.48E-05
**SNP_A-2187715**	10	21219928	A	G	0.4325	0.5865	intronic	NEBL	-0.06329	3.52E-05
**rs6999260**	8	14554773	T	C	0.2863	0.9581	intronic	SGCZ	0.0701	3.60E-05
**rs2833805**	21	33796200	C	T	0.3192	0.08696	intronic	EVA1C	-0.0697	3.82E-05
**SNP_A-1870278**	3	151044732	T	C	0.3382	0.2725	UTR3	P2RY13(NM_176894:c.*1047A>G)	-0.06594	4.15E-05
**rs28366274**	6	32560107	T	G	0.07578	0.3902	intergenic	HLA-DRB1(dist=2494),HLA-DQA1(dist=45076)	0.2167	4.22E-05
**rs857421**	6	14649283	T	C	0.1909	0.8323	intergenic	LINC01108(dist=363598),JARID2(dist=596923)	-0.08179	4.32E-05
**rs10825506**	10	57181541	C	T	0.06463	0.1554	intergenic	PCDH15(dist=620490),MTRNR2L5(dist=177209)	0.1277	4.35E-05

Supplementary Table S12. Genome-wide association analysis results of annual change rate ($\widehat{\beta}_1$) for lung function.

Trait	SNP	CHR	BP	A1	A2	MAF	HWE_P	REGION	GENE	BETA	P
post-FEV_1/FVC (%)	rs2445936	11	117284577	C	T	0.2296	0.8071	downstream	CEP164	0.1573	3.20E-08

Only the variants at genome-wide significant level of 5.0E-08 are listed.

Supplementary Table S13. Genome-wide association analysis results of annual change rate ($\widehat{\beta}_1$) for post-FEV$_1$/FVC. Top 40 variants are listed after clumping.

SNP	CHR	BP	A1	A2	MAF	HWE_P	FUNCTION	GENE	BETA	P
**rs2445936**	11	117284577	C	T	0.2296	0.8071	downstream	CEP164	0.1573	3.20E-08
**rs80070842**	10	108524046	C	T	0.05125	0.911	intronic	SORCS1	-0.2499	3.08E-06
**rs609272**	11	117201682	T	C	0.2593	0.9328	intronic	CEP164	0.1279	3.83E-06
**rs4764465**	12	19325627	T	G	0.1093	0.04764	intronic	PLEKHA5	-0.1798	3.98E-06
**rs9576691**	13	39581230	T	A	0.2037	0.2421	intergenic	STOML3(dist=16234),PROSER1(dist=2772)	-0.1345	7.06E-06
**rs11187149**	10	94485763	G	A	0.2305	0.8797	intergenic	HHEX(dist=30355),EXOC6(dist=108707)	-0.1284	8.89E-06
**SNP_A-2112277**	6	168573159	G	A	0.3696	0.7138	intergenic	FRMD1(dist=93320),LOC101929420(dist=70459)	0.1094	9.81E-06
**SNP_A-4213610**	10	19809674	A	C	0.1966	0.499	intronic	MALRD1	-0.1288	1.82E-05
**rs9394085**	6	32288690	A	G	0.08608	0.534	intronic	C6orf10	0.1859	2.22E-05
**rs2147890**	10	68830365	T	C	0.2499	0.6075	intronic	CTNNA3,LRRTM3	0.117	2.48E-05
**SNP_A-2221645**	5	1300584	G	C	0.2482	1	intergenic	TERT(dist=5422),MIR4457(dist=8841)	-0.117	2.49E-05
**rs4277739**	4	30671050	C	A	0.3797	0.02725	intergenic	MIR4275(dist=1849760),PCDH7(dist=50980)	-0.1072	2.55E-05
**rs113296056**	10	107803909	C	T	0.09967	0.4032	intergenic	LOC101927549(dist=223818),SORCS1(dist=529512)	-0.166	2.56E-05
**rs12606621**	18	75207761	G	C	0.3144	0.3807	intergenic	GALR1(dist=225665),LINC01029(dist=475492)	0.1093	3.01E-05
**rs10404037**	19	30669271	G	A	0.4725	0.8957	intergenic	URI1(dist=161752),ZNF536(dist=194029)	0.1008	3.34E-05
**SNP_A-2041670**	4	38384211	T	C	0.054	0.1172	intergenic	TBC1D1(dist=243415),LINC01258(dist=38072)	-0.2179	3.70E-05
**rs73310474**	10	92980773	T	C	0.3634	0.9623	intronic	PCGF5	0.1053	3.88E-05
**rs9505345**	6	8044803	G	A	0.05041	0.7366	ncRNA_intronic	BLOC1S5-TXNDC5,EEF1E1-BLOC1S5	-0.2239	4.00E-05
**SNP_A-4220421**	12	67909350	G	A	0.1576	0.2275	intergenic	CAND1(dist=200878),LOC100507175(dist=4512)	-0.1351	4.07E-05
**rs12649107**	4	128104744	T	C	0.2756	0.1853	intergenic	MIR2054(dist=1676282),INTU(dist=449343)	-0.1124	4.71E-05
**rs13005182**	2	137575997	T	C	0.3691	0.2082	intronic	THSD7B	-0.1009	5.10E-05
**rs7121034**	11	44827283	A	G	0.0903	0.3526	intronic	TSPAN18	-0.1763	5.14E-05
**rs73984611**	17	32105052	T	G	0.1869	0.9437	intronic	ASIC2	-0.1243	5.19E-05
**rs62322228**	4	127746332	T	C	0.2811	0.123	intergenic	MIR2054(dist=1317870),INTU(dist=807755)	0.105	5.51E-05
**rs2113687**	15	66311045	C	G	0.3441	0.02452	intronic	MEGF11	0.1038	5.68E-05
**SNP_A-1997844**	9	121031029	G	A	0.1762	0.003221	intergenic	TLR4(dist=551260),BRINP1(dist=897879)	-0.129	5.70E-05
**rs11214627**	11	113373404	T	C	0.2569	0.4666	intergenic	DRD2(dist=27403),TMPRSS5(dist=184864)	0.11	6.55E-05
**SNP_A-1929144**	2	137596420	A	T	0.2644	0.2722	intronic	THSD7B	-0.1067	6.72E-05
**rs73406419**	15	48608125	C	A	0.05419	0.4562	intergenic	SLC12A1(dist=11850),DUT(dist=15496)	-0.2113	6.79E-05
**rs62306208**	4	61320615	C	T	0.118	0.05508	intergenic	NONE(dist=NONE),MIR548AG1(dist=467722)	0.1497	6.86E-05
**rs6942008**	6	77991734	G	T	0.4025	0.6553	intergenic	IMPG1(dist=1209339),HTR1B(dist=178831)	0.09784	7.18E-05
**rs8014568**	14	23843363	T	C	0.2678	0.5084	intronic	IL25	0.1074	7.35E-05
**SNP_A-1911103**	11	126527765	A	G	0.4487	0.01387	ncRNA_intronic	LOC101929427	0.09471	7.86E-05
**rs4952177**	2	30921528	G	A	0.2924	0.8147	intergenic	LCLAT1(dist=54437),CAPN13(dist=24110)	-0.1032	7.98E-05
**rs7803087**	7	7868309	T	A	0.1259	0.4603	intronic	UMAD1	0.1407	8.04E-05
**rs60926188**	12	80440185	C	T	0.1779	0.2627	intergenic	PPP1R12A(dist=110950),OTOGL(dist=163048)	-0.1273	8.25E-05
**rs2973418**	5	120570786	G	A	0.3509	0.9237	intergenic	PRR16(dist=547761),LOC102467226(dist=87459)	0.1005	8.35E-05
**rs73934959**	2	69698772	T	A	0.1733	0.6799	UTR3	AAK1(NM_014911:c.*4229A>T)	0.1246	8.74E-05
**rs74761525**	11	117225982	T	C	0.2029	0.9732	intronic	CEP164	-0.1171	8.75E-05
**rs6563663**	13	39559430	C	T	0.3575	0.2421	intronic	STOML3	-0.0997	8.77E-05

Supplementary Table S14. The fixed effects estimations used in SNP the heritability estimation models of subject-specific means (h_0^2) and annual change rates (h_1^2).

Trait	Subject-specific means						Annual change rates					
	Sex	s.e.	Age	s.e.	Height	s.e.	Sex	s.e.	Age	s.e.	Height	s.e.
**FVC (L)**	-0.030	0.001	0.060	0.060	0.609	0.020	-0.017	0.002	-0.016	0.002	0.030	0.038
**FEV_1 (L)**	-0.046	0.001	0.053	0.001	0.484	0.022	-0.008	0.001	-0.020	0.002	-0.231	0.037
**FEV_1/FVC (%)**	-0.044	0.001	-0.021	0.002	-0.396	0.034	-0.003	0.002	0.000	0.002	-0.304	0.038
**FEF25-75% (L/sec)**	-0.058	0.001	0.017	0.002	0.043	0.032	0.010	0.001	-0.011	0.002	-0.274	0.038
**MVV (L/min)**	-0.052	0.001	0.035	0.001	0.648	0.024	-0.015	0.001	-0.004	0.002	-0.334	0.038
**post-FVC (L)**	-0.030	0.001	0.064	0.002	0.621	0.027	-0.015	0.002	-0.021	0.003	0.101	0.052
**post-FEV_1 (L)**	-0.044	0.001	0.059	0.002	0.556	0.029	-0.004	0.002	-0.027	0.003	-0.028	0.052
**post-FEV_1/FVC (%)**	-0.042	0.002	-0.023	0.003	-0.326	0.048	0.004	0.002	-0.008	0.003	-0.049	0.053

h_0^2, SNP heritability of subject-specific means; h_1^2, SNP heritability of annual change rates; Age: mean age at the observed time points. Height: mean height at the observed time points. **s.e,** standard error; FVC, forced vital capacity; FEV_1, forced expiratory volume in one second; FEF 25–75, average forced expiratory flow during the mid (25–75%) portion of the FVC; MVV maximal voluntary ventilation; post-FVC, post-bronchodilator test for FVC; post- FEV_1, post-bronchodilator test for FEV_1; post- FEV_1/FVC, post-bronchodilator test for FEV_1/ FVC ratio

Supplementary Figure S1. The illustrative example of h_0^2 and h_1^2. If we assume that the effect of the environment is small and phenotypes are mostly determined by genetic components. Then phenotypic distributions according to age can differ by h_0^2 and h_1^2. We say Sub_1 and Sub_2 are subject 1 and subject 2.

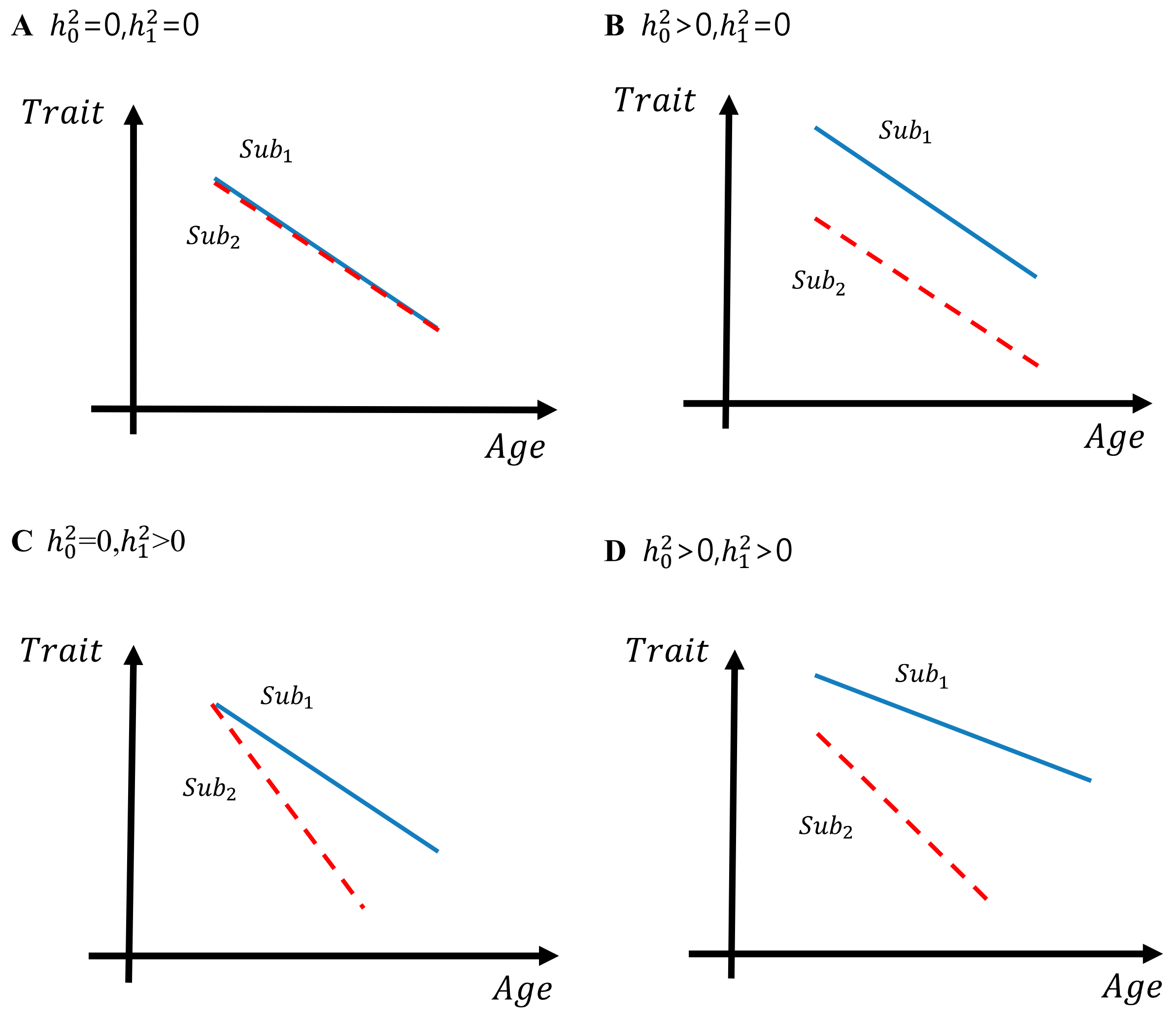

Supplementary Figure S2. Cross-sectional heritability estimates. Heritability estimates of cross-sectionally observed lung functions at different time points and h_0^2 were compared. The solid dot indicates the heritability of cross-sectional measurements at each time point. Black straight and dashed lines indicate their means and medians. Red straight lines indicate h_0^2.

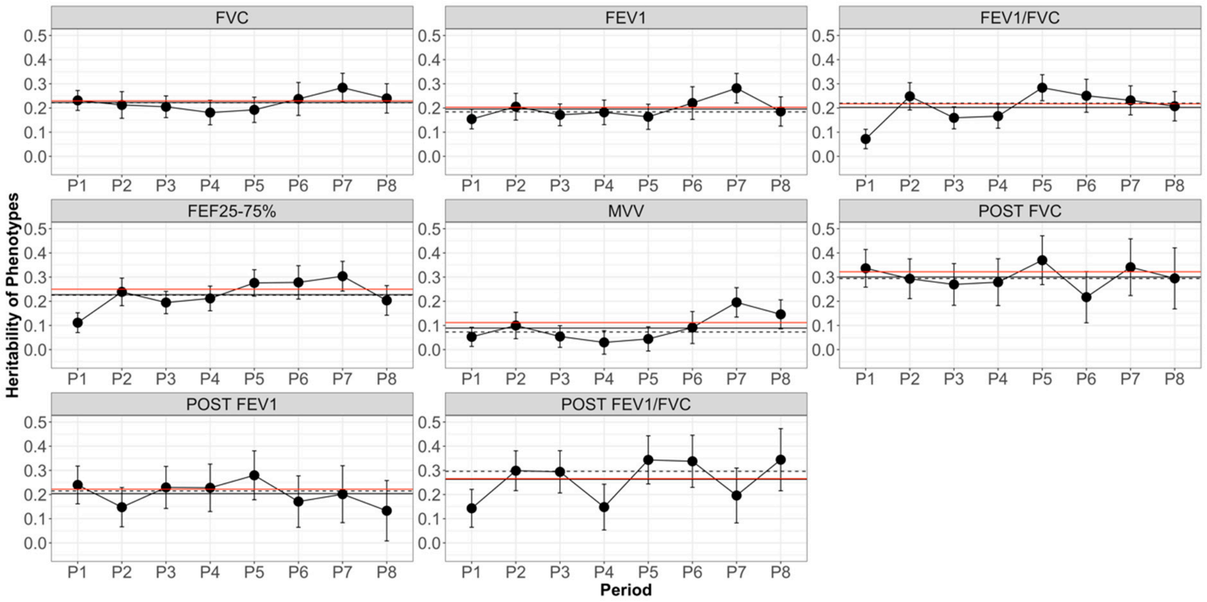

Supplementary Figure S3. (A) Scatter plots between subject-specific mean ($\hat{\beta}_0$) and annual change rate ($\hat{\beta}_1$). (B) Scatter plots between annual change rate rates ($\hat{\beta}_1$) and observed values. Both scatter plots were provided for FEV_1/FVC and post- FEV_1/FVC.

A

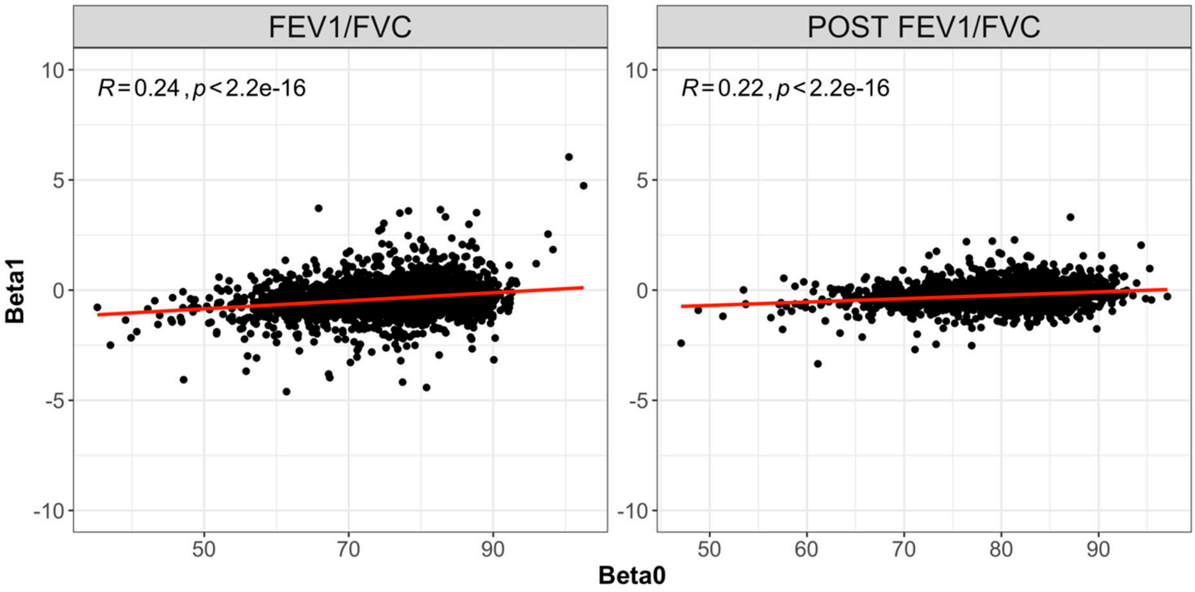

B

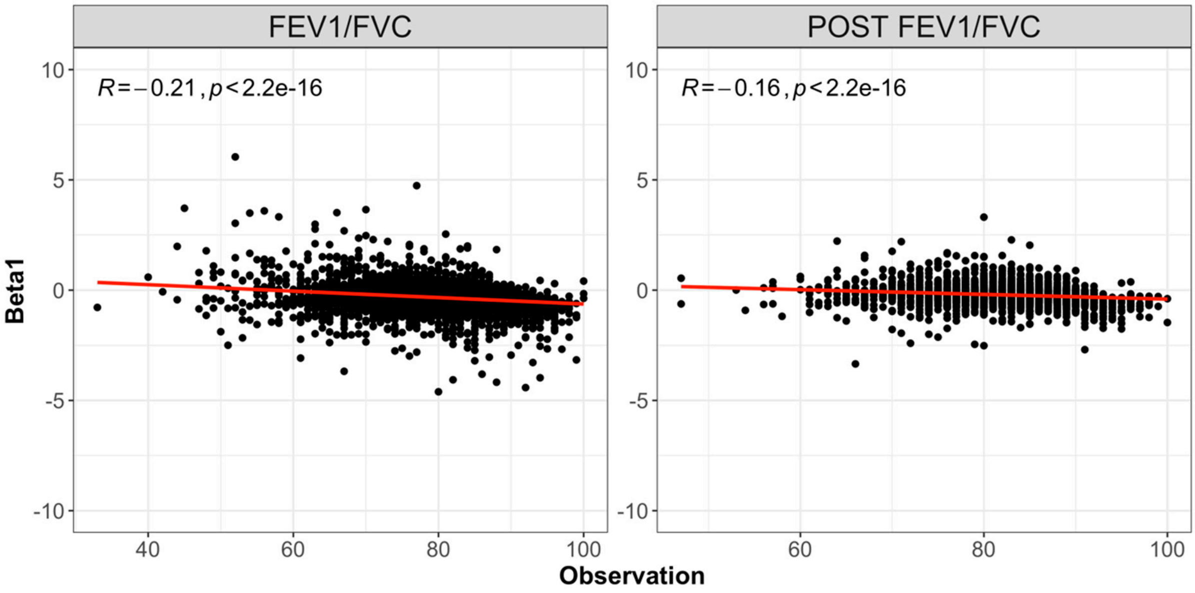

Supplementary Figure S4. Manhattan **Plot and Q-Q plot of subject-specific means ($\widehat{\beta}_0$) for (A) FEV_1, (B) FEV_1/FVC.** Red line indicates genome-wide significant level 5×10^{-8}, and blue line indicates suggestive level 5×10^{-5}. λ is genomic inflation factor.

(A) FEV_1

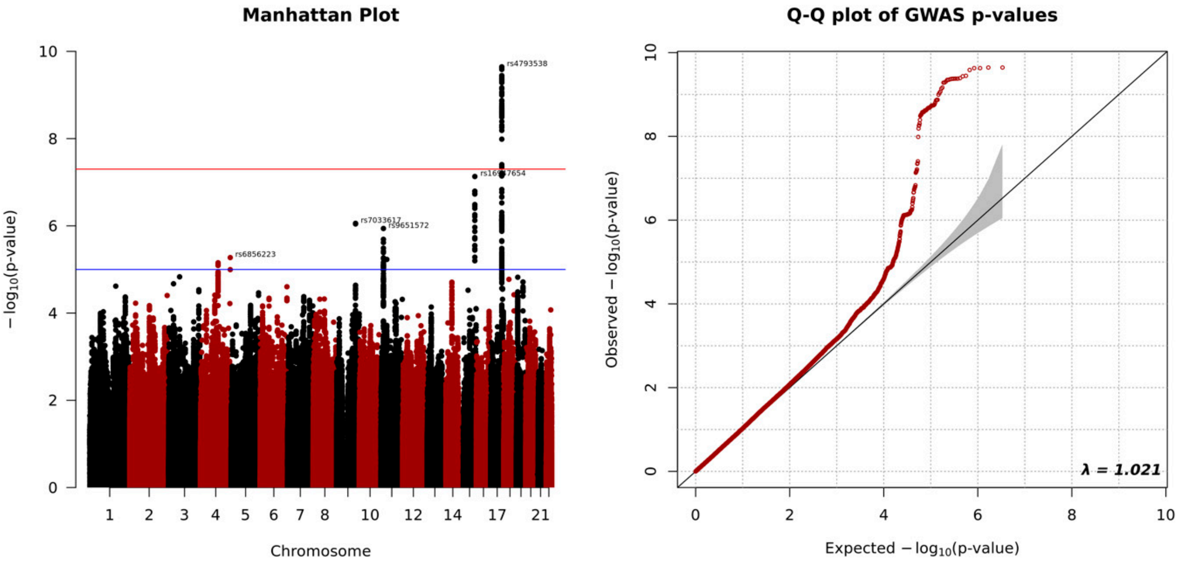

(B) FEV1/FVC

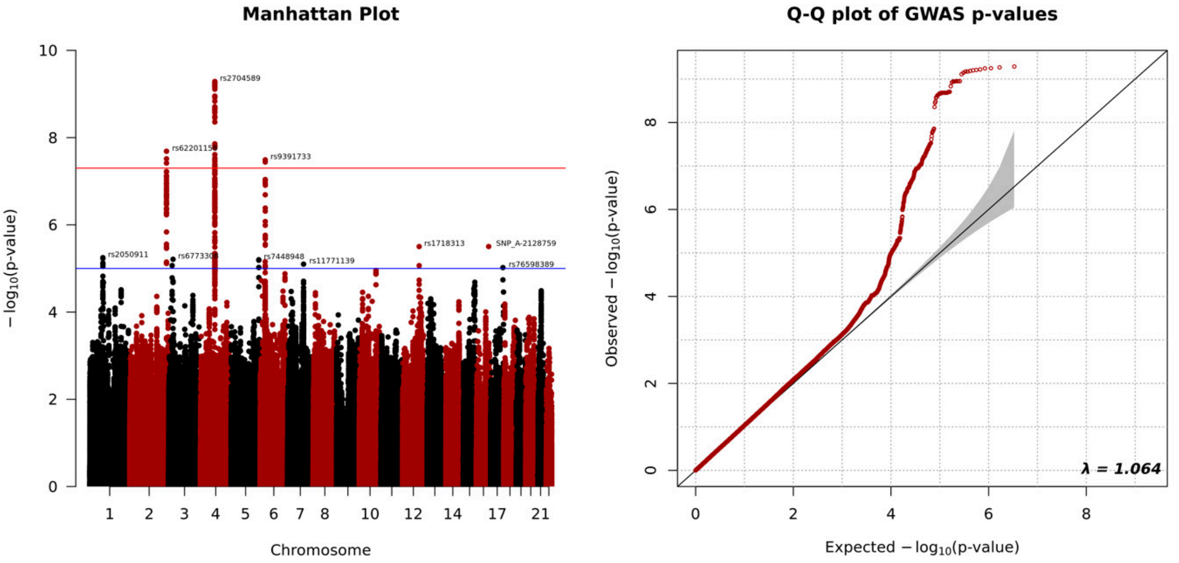

Supplementary Figure S5. Manhattan Plot and Q-Q plot of annual change rates ($\hat{\boldsymbol{\beta}}_1$) for (A) post- FEV$_1$/FVC. Red line indicates genome-wide significant level 5×10^{-8}, and blue line indicates suggestive level 5×10^{-5}. λ is genomic inflation factor.

(A) post-FEV$_1$/FVC

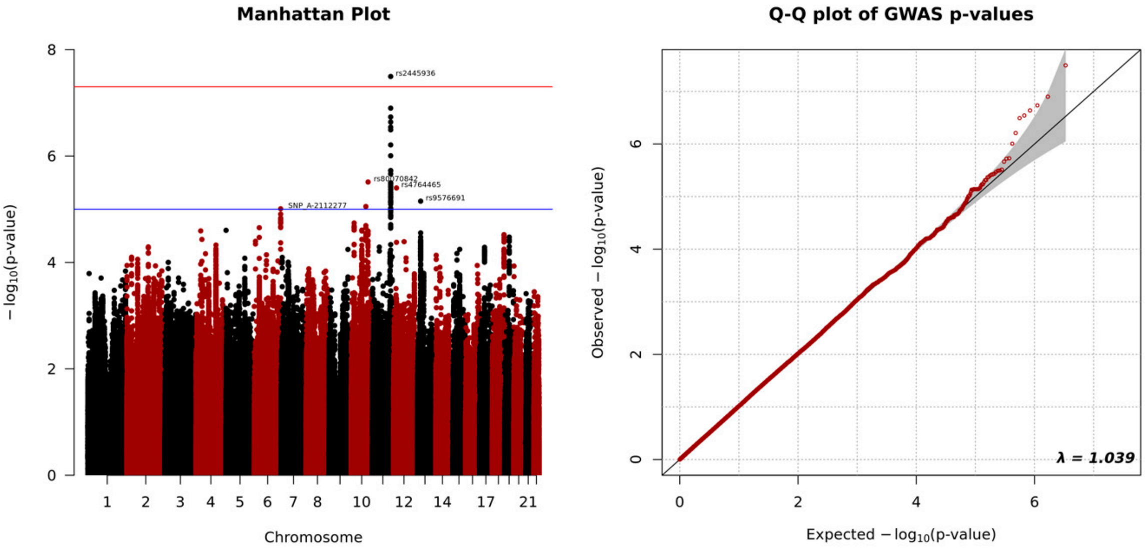

Supplementary Figure S6. Plot of principle component which based one genomic relationship matrix (GRM). The plot showed that all the subjects from the two different cohorts were almost mix together without clear clusters which indicates these subjects were from similar population.

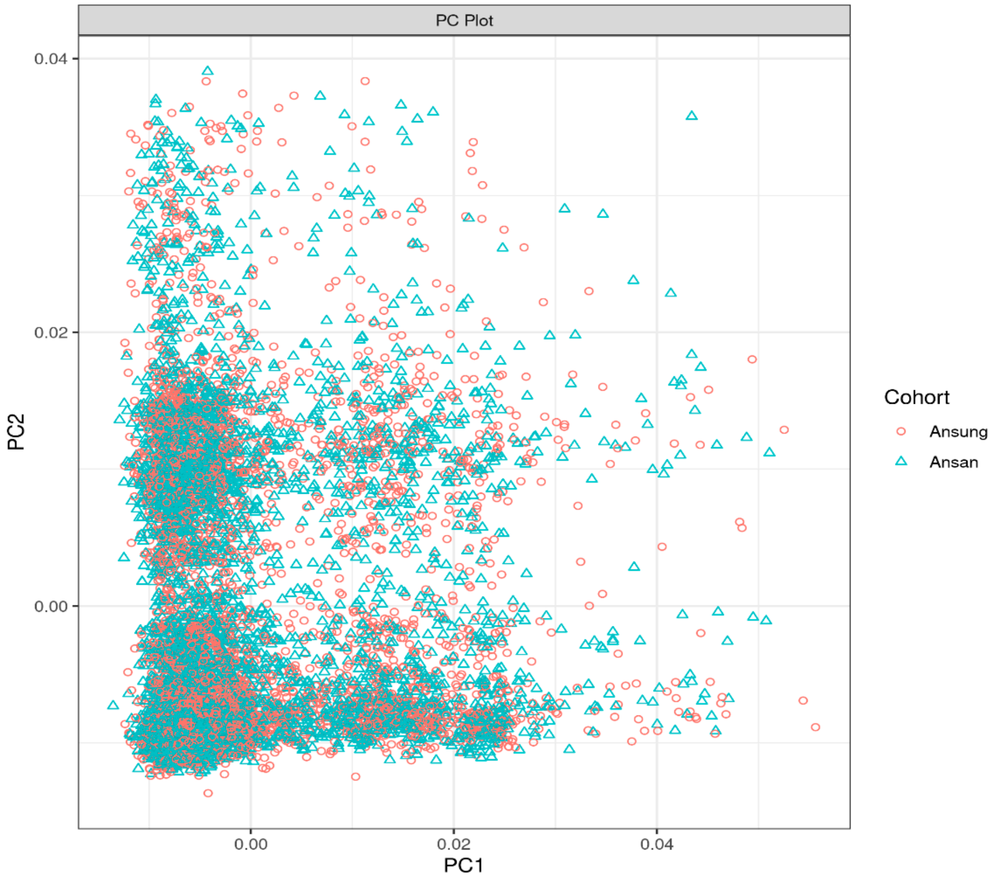

REFERENCES

1. Purcell S, Neale B, Todd-Brown K, Thomas L, Ferreira MA, Bender D, Maller J, Sklar P, de Bakker PI, Daly MJ *et al*: **PLINK: a tool set for whole-genome association and population-based linkage analyses**. *Am J Hum Genet* 2007, **81**(3):559-575.
2. Song YE, Lee S, Park K, Elston RC, Yang HJ, Won S: **ONETOOL for the analysis of family-based big data**. *Bioinformatics* 2018, **34**(16):2851-2853.
3. Delaneau O, Marchini J, Zagury JF: **A linear complexity phasing method for thousands of genomes**. *Nat Methods* 2011, **9**(2):179-181.
4. Howie BN, Donnelly P, Marchini J: **A flexible and accurate genotype imputation method for the next generation of genome-wide association studies**. *PLoS Genet* 2009, **5**(6):e1000529.
5. Howie B, Marchini J, Stephens M: **Genotype imputation with thousands of genomes**. *G3 (Bethesda)* 2011, **1**(6):457-470.
6. Lee SH, Yang J, Goddard ME, Visscher PM, Wray NR: **Estimation of pleiotropy between complex diseases using single-nucleotide polymorphism-derived genomic relationships and restricted maximum likelihood**. *Bioinformatics* 2012, **28**(19):2540-2542.
7. Yang J, Benyamin B, McEvoy BP, Gordon S, Henders AK, Nyholt DR, Madden PA, Heath AC, Martin NG, Montgomery GW *et al*: **Common SNPs explain a large proportion of the heritability for human height**. *Nat Genet* 2010, **42**(7):565-569.
